# Yeast Silent Mating Type Loci Form Heterochromatic Clusters through Silencer Protein-Dependent Long-Range Interactions

**DOI:** 10.1371/journal.pgen.1000478

**Published:** 2009-05-08

**Authors:** Adriana Miele, Kerstin Bystricky, Job Dekker

**Affiliations:** 1 Program in Gene Function and Expression, University of Massachusetts Medical School, Worcester, Massachusetts, United States of America; 2 Department of Biochemistry and Molecular Pharmacology, University of Massachusetts Medical School, Worcester, Massachusetts, United States of America; 3 Laboratoire de Biologie Moléculaire Eucaryote (LBME), University of Toulouse, Toulouse, France; 4 UMR5099, Centre National de la Recherche Scientifique, IFR109, Toulouse, France; Medical Research Council Human Genetics Unit, United Kingdom

## Abstract

The organization of eukaryotic genomes is characterized by the presence of distinct euchromatic and heterochromatic sub-nuclear compartments. In *Saccharomyces cerevisiae* heterochromatic loci, including telomeres and silent mating type loci, form clusters at the nuclear periphery. We have employed live cell 3-D imaging and chromosome conformation capture (3C) to determine the contribution of nuclear positioning and heterochromatic factors in mediating associations of the silent mating type loci. We identify specific long-range interactions between *HML* and *HMR* that are dependent upon silencing proteins Sir2p, Sir3p, and Sir4p as well as Sir1p and Esc2p, two proteins involved in establishment of silencing. Although clustering of these loci frequently occurs near the nuclear periphery, colocalization can occur equally at more internal positions and is not affected in strains deleted for membrane anchoring proteins yKu70p and Esc1p. In addition, appropriate nucleosome assembly plays a role, as deletion of *ASF1* or combined disruption of the CAF-1 and HIR complexes abolishes the *HML*-*HMR* interaction. Further, silencer proteins are required for clustering, but complete loss of clustering in *asf1* and *esc2* mutants had only minor effects on silencing. Our results indicate that formation of heterochromatic clusters depends on correctly assembled heterochromatin at the silent loci and, in addition, identify an Asf1p-, Esc2p-, and Sir1p-dependent step in heterochromatin formation that is not essential for gene silencing but is required for long-range interactions.

## Author Summary

Chromosomes are non-randomly positioned inside cells, and this organization is relevant for genome regulation. Spatial clustering of heterochromatic loci provides a striking example of nuclear compartmentalization. In *S. cerevisiae*, the presence of heterochromatic sub-nuclear domains has been well established, but their mechanisms of formation are not fully understood. Here, we analyzed the DNA elements and protein complexes that are critical for formation of heterochromatic clusters. We focused on heterochromatic regions on chromosome III—the two telomeres, as well as the silent mating type loci *HML* and *HMR*, located on the left and right end of the chromosome, respectively. We employed live cell 3-D imaging and chromosome conformation capture (3C) and found that these loci specifically interact most prominently near silencer elements that flank the loci. Analysis of a panel of mutants showed that complexes involved in silencing are also involved in long-range interactions. Interestingly, we find that heterochromatic interactions are mechanistically distinct from silencing and independent of tethering to the nuclear periphery. Our results indicate that formation of heterochromatic clusters depends on correctly assembled heterochromatin, and point to a step in heterochromatin formation that is not essential for gene silencing but is required for long-range interactions between heterochromatic loci.

## Introduction

The eukaryotic nucleus tends to be organized so that active and inactive sub-nuclear domains are spatially separated [1]–[4]. For instance, active genes co-localize in a limited number of transcription factories, while heterochromatic regions are found clustered in silenced nuclear compartments. Examples of the latter are found in *Drosophila melanogaster* and *Arabidopsis thaliana* where the large heterochromatic regions encompassing the centromeres associate to form a single “chromocenter”, and in mammalian cells where centromeres cluster in a small number of foci [5]–[7]. In most cases heterochromatin is found clustered near the nuclear envelope [1],[8],[9]. In the yeast *Saccharomyces cerevisiae*, heterochromatin is found at and near the 32 telomeres, and at the two silent mating type loci, *HML* and *HMR*, located near the left and right telomere of chromosome III, respectively [10],[11]. These 34 loci co-localize in 4–8 clusters at the nuclear periphery [12]–[16]. A similar phenomenon is observed in *Schizosaccharomyces pombe* in which the heterochromatic centromeres, telomeres, and mating type loci cluster in silent foci at the nuclear periphery [17]. Heterochromatic clusters are thought to represent nuclear sub-compartments that are enriched in silencing proteins, while the rest of the nucleus is depleted in such factors [14],[18],[19]. Although the importance of association of genes with silent compartments in the process of silencing is well established, the mechanisms that drive these interactions are poorly understood.

Formation of heterochromatin at *HM* loci has been characterized in detail (for reviews see [11],[20],[21]). Silencing at *HML* and *HMR* requires cis-acting silencer elements [11]. Protein complexes, such as Rap1p and the Origin Recognition Complex (ORC), bind to these silencer elements and help recruit Silent Information Regulator (Sir) proteins. Sir1p associates with Orc1p. Subsequently, Sir4p is recruited to the silencers via its interaction with Rap1p and Sir1p. Sir4p likely recruits Sir2p and is also required to recruit Sir3p to the silencer. Sir2p is a NAD-dependent histone deacetylase that deacetylates H4 K16 at nearby nucleosomes, which provides a binding site for additional SIR2-4 complexes [22],[23]. This positive feedback loop allows spreading of the SIR2-4 complex throughout the mating type loci, resulting in positioned nucleosomes and gene silencing throughout the region [24],[25]. Thus, histones and appropriate nucleosome assembly contribute to formation of heterochromatin, perhaps due to the fact that binding and spreading of the Sir complex occurs through direct interactions with histones. In addition, genetic evidence indicates that the histone chaperone Asf1p and the CAF-1 and HIR nucleosome assembly complexes have partially overlapping functions in heterochromatin formation [26],[27]. Finally, previous work has indicated that silencer elements can cooperatively silence *HMR* in a manner that may involve direct interactions between *HMR-E* and *HMR-I* [28].

The clustering of Sir-bound loci in a limited number of sub-nuclear domains has been used as a model to study the processes that drive nuclear compartmentalization. Formation of silent nuclear compartments results in limiting Sir proteins to only a small number of locations in the nucleus [14]. In that situation, only loci located in these compartments will have access to silencer proteins and become heterochromatic, thereby preventing SIR complex-mediated silencing at inappropriate locations.

A major unanswered question is how compartmentalization is established and maintained. Is clustering of loci driven by association of individual loci to a common sub-nuclear structure, e.g. sites on the nuclear envelope, or is clustering an intrinsic property of heterochromatin that depends on local assembly of silencing complexes at these loci? Answers to this question can have important implications for our understanding of causal relationships between nuclear organization and gene regulation. To address this issue we have used chromosome III as a model for clustering of silent loci. This chromosome contains 4 heterochromatic loci: two telomeres and the nearby silent mating type loci, *HML* and *HMR*. We employ live cell 3D imaging to show that *HML* and *HMR* frequently co-localize both at the nuclear periphery as well as at more internal locations of the nucleus, indicating that anchoring to the nuclear envelope (NE) is not required for *HML*-*HMR* interactions. Using chromosome conformation capture (3C) [29] we find that *HML* and *HMR* frequently and specifically interact with each other. Interactions are most frequent around the *HML-E* and *HMR-I* silencers. Analysis of a series of mutants reveals that clustering of these loci critically depends on silencer proteins, but that it is independent of proteins that contribute to anchoring silent loci at the nuclear periphery. Furthermore, silencing is not sufficient for the *HM* loci interaction to occur. Based on these observations we propose that silent compartments are not pre-assembled to facilitate subsequent recruitment of heterochromatic proteins. Instead we propose that long-range interactions between *HM* loci depend on a particular step in local heterochromatin assembly, which requires at least Asf1p, Esc2p and Sir1p.

## Results

**Figure 1:**
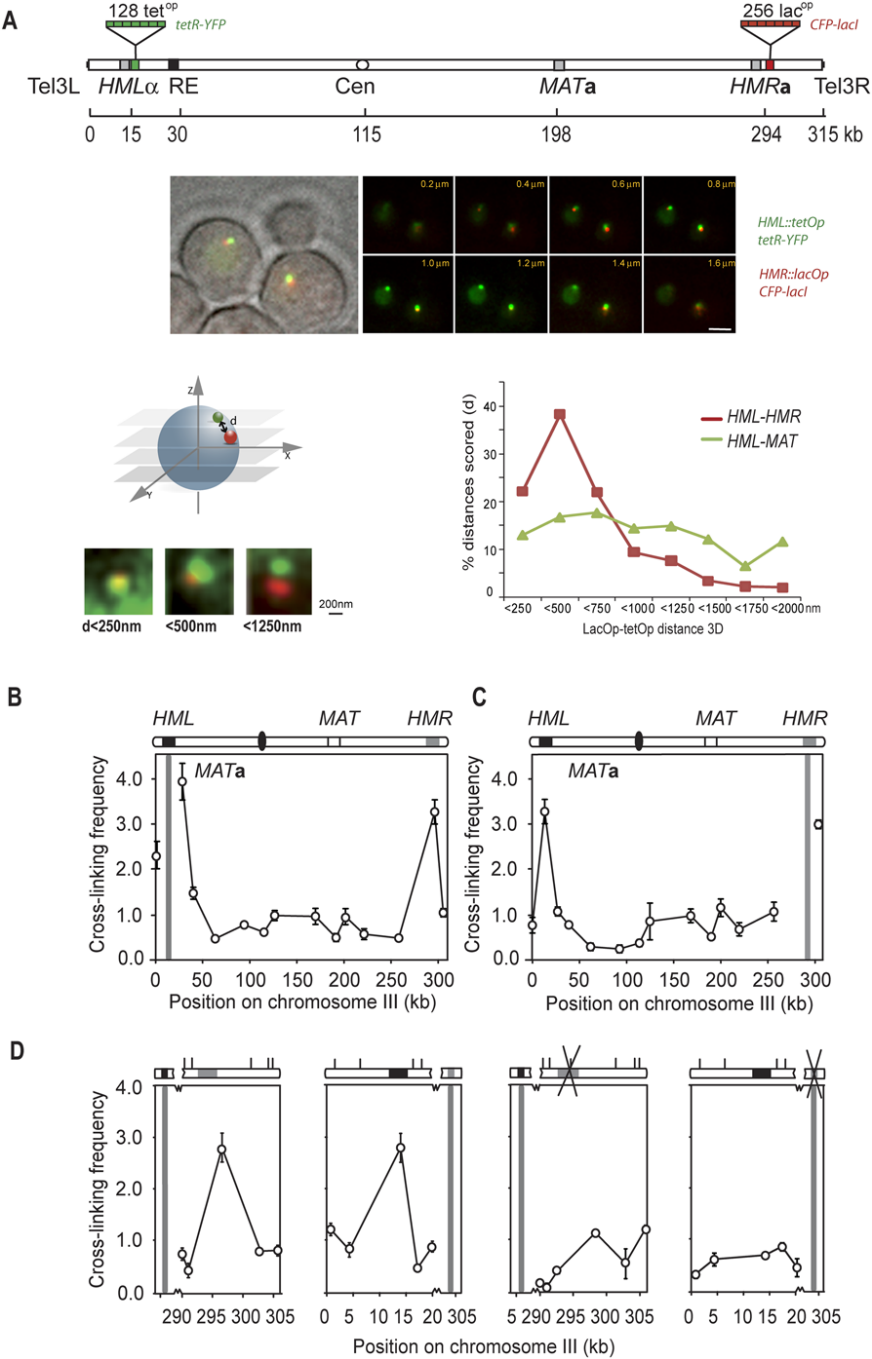
Long-range interaction between *HML* and *HMR*. (A) 3D live cell analysis of *HML-HMR* colocalization in wild-type yeast during interphase (G1 and S combined). Image stacks were acquired at 0.2 µm spacing along the z-axis of yeast strains having targeted integration of lac^op^ and tet^op^ arrays and expressing LacI-CFP and TetR-YFP. Shown are fluorescent images of insertion sites near *HML* and *HMR* of the 8 most central focal planes at the indicated z distance from the bottom plane. The distributions of 3D measurements are plotted in incremental 250 nm categories. *Tet* ^op^ and *lac* ^op^ insertion sites within unique sequences distal to *HML*, *HMR* or proximal to *MAT* loci are schematically represented. Bar is 2 µm. (B) 3C analysis of chromosome III in *MAT* *a* wild-type yeast strains. The chromosome depiction portrays *HML* as a black rectangle, *HMR* as a gray rectangle, and the black oval as the centromere. Crosslinking frequencies are plotted at the midpoint of each restriction fragment. Error bars indicate standard error of the mean (n = 3). Analysis of interactions in *MAT* *a* cells examining crosslinking frequencies between the *Eco*RI fragment containing *HML* (indicated by vertical grey bar) with other restriction fragments along the length of chromosome III. (C) Analysis of interactions in *MAT* *a* cells examining crosslinking frequencies between the *Eco*RI fragment containing *HMR* (indicated by vertical grey bar) with other restriction fragments along the length of chromosome III. (D) Fine mapping 3C analysis of chromosome III in *MAT* *a* cells (left two panels) and *MATα hmr*Δ cells (right two panels). 1^st^ panel: Analysis of interactions in *MAT* *a* cells between the *Eco*RI fragment containing *HML* with restriction fragments immediately flanking *HMR*. 2^nd^ panel: Analysis of interactions in *MAT* *a* cells between the *Eco*RI fragment containing *HMR* with restriction fragments immediately flanking *HML*. 3^rd^ panel: Analysis of interactions in *MATα hmr*Δ cells between the *Eco*RI fragment containing *HML* with restriction fragments immediately flanking the *kanMX4* cassette. 4^th^ panel: Analysis of interactions in *MAT*α *hmr*Δ cells between the *Eco*RI fragment containing the *kanMX4* cassette with restriction fragments immediately flanking *HML*. Sites marked with an ‘x’ have been deleted from the strain.

Previous studies have shown that the ends of chromosome III are relatively close in three-dimensional space in the yeast nucleus [29],[30]. To explore the spatial relationship of *HML* and *HMR in vivo* we have differentially tagged the two ends of chromosome III using repetitions of the *tet* ^op^ and *lac* ^op^ sequences inserted within unique sequences directly adjacent to *HML* or *HMR* respectively (Figure 1A). Insertions are visualized by the binding of YFP- or CFP-fusions to the corresponding bacterial repressors. We extended previous studies, which used this same strain to analyze *HML-HMR* distances in G1 cells only [30], to all interphase cells so that the distance data could be directly correlated with 3C analyses of whole yeast cultures described below. Distances between the resulting fluorescent spots were measured on 3D stacks of intact cells in interphase (Figure 1A). The distributions of 3D measurements are plotted in incremental 250 nm categories. In 21% of wt cells scored (n = 836), the distance between *HML* and *HMR* is less than 250 nm indicating that *HML* and *HMR* specifically colocalize in a large number of cells. In an additional 38% of nuclei, the ends of chromosome III are juxtaposed with less than 500 nm separating the fluorescent spots. The mean distance scored was 531 nm. We note that colocalization frequencies reported here for cells in interphase are comparable to data in G1 cells alone where in 39% of cells scored the distance between *HML* and *HMR* was less than 400 nm [30]. Previous work on telomere positioning had identified differences between G1 and S-phase (e.g. [16]). The fact that we obtained co-localization values in exponentially growing cultures that are comparable to those previously measured in G1 is likely related to the fact that most cells in our cultures are in G1 (>70%).

As a control we assessed the 3D distance between *tet* ^op^ and *lac* ^op^ insertion sites distal to *HML* and proximal to *MAT* respectively. The genomic distance between these sites located on opposite arms of chromosome III is 177 kb versus 280 kb for the sites near *HML* and *HMR*. In this strain *HML* and *MAT* colocalized in only 12% of cells scored (Figure 1A; n = 214) in agreement with the observation by Houston and Broach [31] that *HML* and *MAT* regularly but transiently contact each other in the absence of HO-mediated cleavage at *MAT*. Importantly, more than 70% of distances were larger than 500 nm (mean distance 916 nm).

We conclude that although the loci are clearly not co-localized in all cells at all times, the high frequency of colocalization between *HML* and *HMR*, but not between *HML* and *MAT* in intact interphase cells shows that *HML* and *HMR* are preferentially juxtaposed.

### 3C Analysis of *HML*-*HMR* Association

Next, we employed chromosome conformation capture (3C) to further analyze co-localization of the heterochromatic loci *HML* and *HMR* in more detail and at higher resolution [29].

Previous 3C analyses of yeast chromosomes used purified nuclei, which may result in loss of some interactions due to the rather disruptive nuclei isolation protocol. Therefore, we adapted 3C for use with intact yeast cells [32]. In this method, the cell wall is removed by zymolyase treatment and intact spheroplasts are treated with formaldehyde to induce cross-links between proteins and DNA and proteins and other proteins thereby trapping interacting chromatin fragments throughout the yeast genome. Cross-linked spheroplasts are then solubilized by SDS and Triton X-100. From here the conventional 3C protocol is followed including restriction digestion, DNA ligation, and reversal of cross-links. The identities of interacting fragments are determined through detection of 3C ligation products by semi-quantitative PCR using locus specific primers. In addition, a randomized ligation control is generated which serves as a control for primer efficiency. This template is generated by digesting purified yeast genomic DNA followed by random intermolecular ligation which results in a DNA sample in which every possible ligation product is present in equal molar amounts.

The cross-linking frequency of two loci is determined by PCR using the 3C and the control libraries as templates. Primers are designed that recognize the corresponding ligation product and PCR products are quantified by ethidium bromide staining of agarose gels. We have found that this quantification method reliably measures the relative abundance of ligation products as long as the PCR is performed within the linear detection range [29], [33]–[36]. Figure S1A and B show examples of determination of the linear range of PCR by titrating the template concentration.

The ratio of the amount of PCR product obtained with the 3C library and the control library is a direct measure (though in arbitrary units) for the frequency with which two loci interact (extensively described in [29],[32],[37],[38]). Each crosslinking frequency is determined in triplicate and averaged. In general, sites that are located close together (within up to 20 kb) will give relatively high 3C crosslinking frequencies while sites that are located far apart will show increasingly lower crosslinking frequencies [29],[33],[38]. Specific long-range interactions between two loci are apparent when their crosslinking frequency is significantly over this background level of interaction [38].

### Specific Interactions between *HML* and *HMR*

We first determined whether *HML* and *HMR* interact more frequently with each other than with other loci on chromosome III. We performed 3C on exponentially growing haploid *MAT* ***a***-cells and determined crosslinking frequencies between the *Eco*RI fragment that contains *HML* and a number of *Eco*RI fragments along the length of chromosome III including the fragment containing *HMR* (Figure 1B). Primer sequences and positions of *Eco*RI restriction fragments are listed in Table S1 and depicted in Figure S1C. As expected, we found that *HML* interacts frequently with sites very close to it and that this crosslinking frequency decreases for restriction fragments located progressively closer to the right arm of the chromosome, similar to what has been observed in other studies (Figure 1B; [29],[33],[34],[36],[38],[39]). Interestingly, we observed a clear peak of crosslinking frequency corresponding to the *Eco*RI fragment containing *HMR*. This indicates that the interaction with *HML* is more frequent than with any other locus in that chromosomal region and thus suggests that the interaction is specific.

To further confirm the interaction between *HML* and *HMR*, we performed the reverse experiment, in which crosslinking frequencies were determined between the *Eco*RI fragment that contains *HMR* and the same *Eco*RI fragments including the fragment containing *HML* (Figure 1B). Again, a peak of crosslinking frequency corresponding to the *Eco*RI fragment containing *HML* is observed. Furthermore, specific and prominent interactions were also determined between *HML* and *HMR* in *MATα* cells (Figure S2A and B), indicating the *HM*-interactions are not specific to one mating type. Further, 3D imaging in *MATα* cells did not reveal a difference in *HML-HMR* interactions, nor did 3C analysis of strains deleted for the Recombination Enhancer [40] (Figure S2 Panels C and D). Given the lack of any mating type specific differences in *HML-HMR* interactions we consider it not likely that any mating type specific proteins or the *MAT* locus itself plays a role in the interaction between silent mating type loci. Interestingly, we do note that there appears to be a loss of frequent interactions between *HML* and sites close to it specifically in *MATα* cells. Although the reason for this is unclear, future comprehensive and chromosome-wide studies can be aimed at analyzing mating type specific differences in the conformation of chromosome III. Here we focused specifically on *HML*-*HMR* interactions, which are unaffected by mating type.

Next we tested whether the *HM* loci also interact with the telomeres of chromosome III. We performed 3C with primers annealing immediately adjacent to the left or to the right telomere (Figure S2E and F). Interactions between the left telomere and other *Eco*RI fragments along chromosome III revealed frequent interactions between the left telomere and *HML* (Figure S2E). This is most likely due to the fact that these loci are located close to each other on the chromosome, which typically results in very frequent background interactions [29],[38]. Interactions along the length of the chromosome between the left telomere and other *Eco*RI fragments were generally lower than observed for *HML* or *HMR*. Interestingly, the left telomere interacted also relatively frequently with *HMR*. The same is true for the right telomere (Figure S2F): this telomere interacted frequently with nearby fragments, including *HMR*, and with *HML*. Although the telomeres interacted preferentially with the *HM* locus located on the opposite side of the chromosome, these interactions are clearly less frequent than the *HML*-*HMR* interaction. Contacts between the two telomeres were also less frequent than the interaction between the *HM* loci (Figure S2F).

3C is used to determine the relative crosslinking frequency in a population of cells, but 3C does not directly reveal the percentage of cells that are engaged in a certain configuration, just like chromatin immunoprecipitation experiments do not provide insight in absolute occupancy levels of proteins at specific loci. Our live cell imaging (Figure 1A) allows direct comparison of 3C crosslinking frequencies to the probability with which loci colocalize in living cells. We find that *HML* and *HMR* are co-localized in 21% of cells and closely juxtaposed in an additional 38% of cells. In contrast *HML* and *MAT* are co-localized in only 12% of cells and closely juxtaposed in another 17%. These different levels of *in vivo* co-localization closely correspond to relative 3C crosslinking frequencies detected for *HML*-*HMR* and *HML*-*MAT* (Figure 1B). Thus, the quantitative agreement between fluorescence microscopy results and data obtained by 3C confirm that the *HM* loci are co-localized in a significant fraction of cells at any given moment. Combined, our results indicate that 3C data provide an accurate proxy for frequency with which loci are closely juxtaposed *in vivo*.

### Preferred Interaction between Regions Containing the *E*- and *I*-Silencers

We wished to firmly rule out the possibility that the observed interaction between the *HM* loci could be indirect and a consequence of contacts between other sub-telomeric elements. We used several approaches. First, we repeated the 3C analysis by including additional primers that detect interactions with *Eco*RI fragments directly flanking *HML* and *HMR* in *MAT* ***a*** cells. We find that the peak of crosslinking frequency of *HML* and *HMR* corresponds to the precise location of these loci and that the crosslinking frequencies decrease dramatically immediately upstream and downstream of the fragment containing the *HM* loci (Figure 1D left two panels). We observed the same in cells of the opposite mating type (*MATα*, not shown).

As a second approach we created a *MATα* strain in which the *HMR* locus was replaced with the *KanMX* cassette. We determined crosslinking frequencies between the fragment containing *HML* and fragments along the length of chromosome III including the *Eco*RI fragments containing and directly flanking the *KanMX* cassette (Figure 1D third panel). We find that the peak of interaction is no longer observed and that the crosslinking frequency of *HML* with the fragments containing the *KanMX* cassette is similar to that of its neighbors. Likewise, when we analyzed interactions between the fragments containing the *KanMX* cassette with fragments along chromosome III including the fragment containing *HML*, we no longer detected the peak of interaction at *HML* (Figure 1D fourth panel). These results indicate that the frequent interaction at *HML* observed in wild type cells requires the presence of *HMR*. In addition, this experiment rules out that the interaction is due to the presence of another genomic element in the sub-telomeric region located outside *HMR* but within the interacting *Eco*RI fragment.

**Figure 2:**
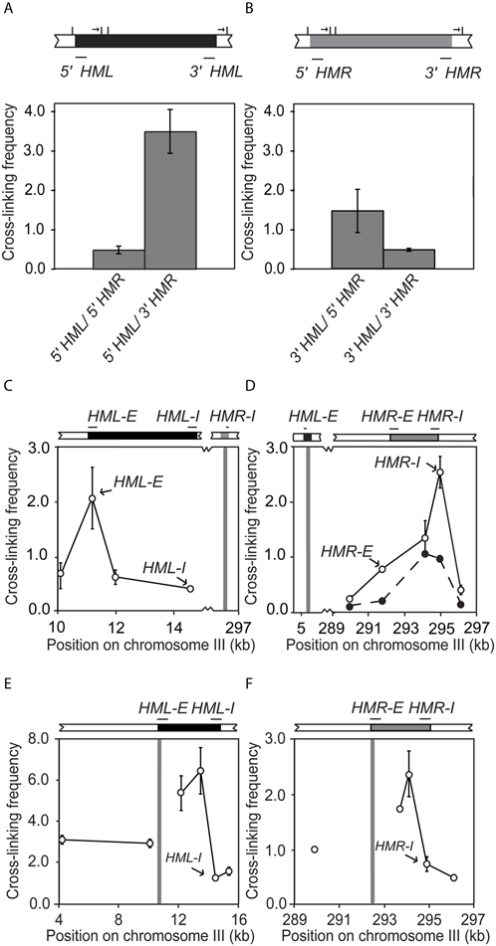
Preferred interaction of *E*- and *I*- silencers from opposite silent loci. Hatch marks represent restriction sites. (A) Crosslinking frequencies between the *Xba*I fragment containing 5′ end of *HML* and either the 5′ end of *HMR* or the 3′ end of *HMR* in *MAT* *a* cells. (B) Crosslinking frequencies between the *Xba*I fragment containing 3′ end of *HML* and either the 5′ end of *HMR* or the 3′ end of *HMR*. (C) Analysis of interactions in *MAT* *a* cells between the *Aci*I fragment containing *HMR-I* (position 294510–295324, primer O52; primer sequences are given in Table S1) with fragments within and immediately outside of *HML*. (D) Analysis of interactions between the *Aci*I fragment containing *HML-E* (position 10815–11636, primer O42) with fragments within and immediately outside of *HMR* (open circles). Analysis of interactions between an *Aci*I fragment within *HML* (position 11679–11906, primer O43) with fragments within and immediately outside of *HMR* (black circles). (E) Analysis of interactions in *MAT* *a* cells between the *Aci*I fragment containing *HML-E* with sites within and immediately outside of *HML*. (F) Analysis of interactions in *MAT* *a* cells between the *Aci*I fragment containing *HMR-E* with sites within and immediately outside of *HMR*.

As a third approach, we determined the role of specific parts of *HML* and *HMR* in mediating their interaction. We analyzed the interaction between the heterochromatic loci using a different restriction enzyme, *Xba*I, as this enzyme cuts inside of *HML* and *HMR* to create fragments in which the left and right ends of *HML* and *HMR* are contained on separate restriction fragments. 3C primer sequences are listed in Table S1. Interestingly, we find that the *Xba*I fragment containing the 5′ end of *HML* interacts preferentially with the fragment containing the 3′ end of *HMR* (Figure 2A). Similarly, the fragment containing the 3′ end of *HML* preferentially interacts with the fragment containing the 5′ end of *HMR* (Figure 2B). Furthermore, the interaction between the 5′ end of *HML* and the 3′ end of *HMR* is clearly the most frequent.

These results point to the possibility that the interaction between *HML* and *HMR* involves the *E*- and *I*-silencer elements that flank *HML* and *HMR* on their 5′ and 3′ side, respectively. To test this we repeated the 3C analysis with a frequently cutting restriction enzyme *Aci*I that cuts at multiple locations within the *HM* loci. We find that the small 814 bp *Aci*I fragment containing the *HMR-I* element (genomic position 294510–295324) most strongly interacts with the 821 bp fragment containing the *HML-E* silencer (genomic position 10815–11636), and significantly less frequently with other parts of *HML* (Figure 2C). Conversely the fragment containing the *HML-E* silencer interacts most prominently with the *HMR-I* fragment, as compared to other regions of *HMR* (Figure 2D). As a control we determined crosslinking frequencies between an *Aci*I fragment located in the middle of *HML*. This fragment (genomic position 11679–11906) interacted most prominently with the *HMR-I* fragment and the neighboring *Aci*I fragment located within *HMR*, but the crosslinking frequencies were lower than that observed between *HML-E* and *HMR-I* (Figure 2D), which is as expected for a fragment located just next to the site of interaction. These results strongly suggest that the *HML-E* and *HMR-I* silencers, or elements located very close to them, are the sites of interaction. Despite intense efforts, we have not been able to generate a mutant in which *HMR-I* was deleted without also creating unexplained rearrangements in the locus. Therefore we cannot unequivocally conclude that the interactions between *HML* and *HMR* are directly mediated by the silencer elements.

Given the interaction observed between regions containing *E*- and *I*-silencers from opposite loci, we then asked whether *E*- and *I*-silencers from the same loci also interact (Figure 2E and 2F). 3C analysis using the *Aci*I enzyme indicates that the *Aci*I fragments containing *HML-E* and *HMR-E* elements interact frequently with nearby sites. Interestingly, *HML-E* and *HMR-E* interacted more strongly with sites within the silent loci than with sites located outside *HML* and *HMR*, despite being separated by comparable genomic distances. One explanation could be that the silent loci are more compact than active chromatin, which can result in increased 3C crosslinking frequencies [33],[34],[36]. Alternatively, *HML-E* and *HMR-E* interact preferentially with a site within *HML* and *HMR* respectively. Interestingly, we note that the peak of interaction is near the promoters of *HMR* ***a*** and *HML*α Importantly, the interactions of *HML-E* and *HMR-E* with *HML-I* and *HMR-I* respectively were significantly less frequent than other interactions throughout these regions (Figure 2E,F). If a loop existed between silencer elements within a given locus these preferred interactions would stand out as peaks on top of a less frequent background of interactions. Our results indicate that although cross-linking frequencies are generally increased within the silent loci, there is no preferential interaction between the *E*- and *I*- silencers of each *HM* locus as compared to interactions with other sites throughout the *HM* loci.

### Sir Proteins Are Required for *HML* and *HMR* Interaction

**Figure 3:**
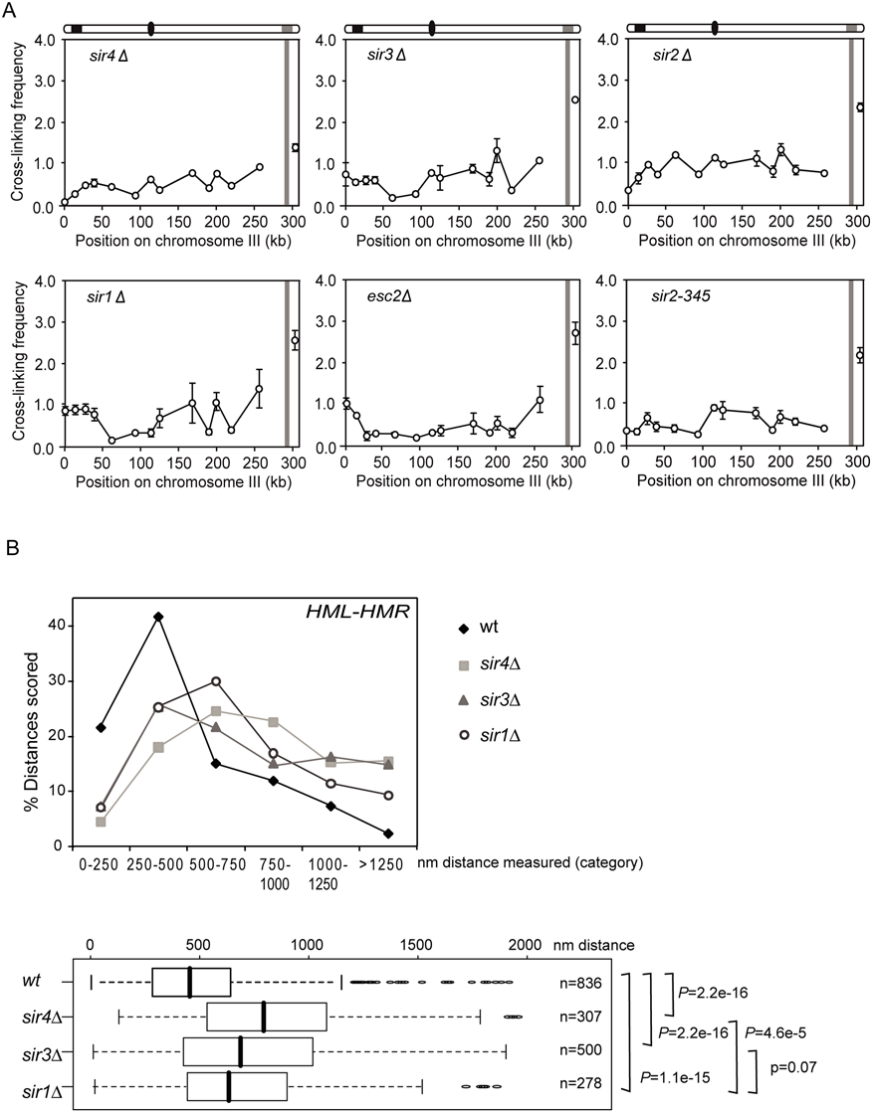
Role of silencing proteins in *HM* interactions. (A) Analysis of interactions in *sir4Δ*, *sir3Δ*, *sir2Δ*, *sir1Δ*, *esc2Δ* and *sir2-345* cells examining crosslinking frequencies between the *Eco*RI fragment containing *HMR* with other restriction fragments along the length of chromosome III. (B) 3D live cell analysis of *HML-HMR* colocalization in *sir4Δ*, *sir3Δ*, *sir2Δ* and *sir1Δ* cells. Image stacks were acquired as in Figure 1 and analyzed by the SpotDistance plug-in of ImageJ. R software was used for the representation of distance distributions as a box plot. Outliers are defined as 1.5 times the Inter Quartile Range (IQR) and are represented as open circles. Distance frequencies were compared using a Wilcox test.

To determine whether *HML* and *HMR* need to be in a heterochromatic state for them to interact, we analyzed mutants that are defective in silencing. We first chose to analyze *sir4Δ*, *sir3Δ* and *sir2Δ* cells, because in these mutants silencing at both *HML* and *HMR* is completely lost [41]. Using 3C, we find that *HMR* and *HML* no longer interact in *sir4*Δ, *sir3*Δ and *sir2*Δ mutant strains (Figure 3A). Interactions between *HMR* and the adjacent right telomere are not affected to a similar extent. Interestingly, the interaction between *HMR* and the right telomere is reduced in the absence of Sir4p but not upon deletion of *SIR2* and *SIR3*. This observation could be related to the degree of peripheral anchoring of these loci (see below).

In agreement with the 3C data, colocalization of *HML* and *HMR*, as determined by live cell fluorescence microscopy, is largely reduced in *sir4*Δ and *sir3Δ* cells, in which only 5% and 7% of distances scored are <250 nm respectively (Figure 3B). As compared to wild-type where in ∼60% of the cells *HML* and *HMR* are found colocalized or adjacent to each other, in *sir4Δ* cells the distance between these loci is less than 500 nm in only 21% of cells (Figure 3B). Similarly, in *sir3Δ* cells <35% of cells scored show *HML* and *HMR* colocalized or immediately adjacent to each other (n = 500; Figure 3B). The distribution of 3D distances measured in *sir3*Δ (n = 500) and *sir4*Δ (n = 307) cells is clearly shifted to greater distances as compared to wt (n = 836). These results indicate that the heterochromatic structure established by the Sir complex or the Sir4p, Sir3p, and Sir2p proteins themselves are critical for the *HML*-*HMR* interaction.

To extend our study, we asked whether Sir1p, a protein that interacts with silencer elements flanking *HML* and *HMR* via the Rap1p/ORC complex during the establishment of the silent chromatin state [42] participates in *HM* loci interactions. Interestingly, we find that, similar to *sir4*Δ, *sir3*Δ, and *sir2*Δ mutants, in *sir1Δ* cells *HMR* and *HML* no longer interact as shown by 3C and by 3D microscopy (Figure 3A,B), despite significant residual silencing (see below). Interactions between each *HM* locus and the telomere on the opposite end of chromosome III are concomitantly reduced (Figure S2G and H).

Given the results obtained with *sir1Δ* cells we chose to determine the role of Esc2p that has been identified as a protein that can functionally substitute for Sir1p [43]. Although it is currently not known whether Esc2p directly binds the *HM* loci, there is strong evidence suggesting that Esc2p is directly affecting the *HM* loci. First, Esc2p was identified as a protein that when targeted to *HML* can induce silencing [44]. Second, Esc2p has been shown to directly bind Sir2p [44]. Third, overproduction of Esc2p can substitute for Sir1p and aids in the establishment of silencing at *HM* loci [43]. Deletion of *ESC2* has only minimal effects on silencing ([43], and see below). Interestingly, as in *sir1Δ* cells, we find that deletion of *ESC2* completely abolished the specific interaction between *HML* and *HMR* (Figure 3A). We conclude that Esc2p plays a critical role in *HML-HMR* interactions, presumably by directly acting on the *HM* loci, although we cannot formally rule out a more indirect role.

Furthermore, given the importance of the silencers and silencing proteins for the *HML* and *HMR* interaction, we chose to analyze a mutant in which silencing proteins are recruited (albeit to a lesser extent than in wild-type) and assembled at the silencers but in which the Sir complex fails to spread across the silenced loci [45]. The mutant *sir2-345* contains a point mutation at residue 345, which results in an Asn-to-Ala substitution. This mutant lacks deacetylase activity which results in a defect in silencing [23]. In a *sir2-345* mutant, an interaction between *HML* and *HMR* can no longer be detected by 3C (Figure 3A). This indicates that in order for this interaction to occur proper heterochromatin must be formed and that the mere presence of Sir proteins at the silencers is not sufficient to promote *HM* loci interaction.

A defect in silencing leads to expression of both **a**- and α- information from *HMR* and *HML* respectively, as in diploid cells (i.e. they display defects in mating). Therefore, we analyzed a diploid strain to determine whether the loss of *HML-HMR* interaction is due to the *sir* mutant cell's diploid characteristics. We found no significant difference in the crosslinking frequencies between *HML* and *HMR* in wild type diploid and haploid strains (Figure S3A). We conclude that the loss of *HML*-*HMR* interactions in *sir4Δ*, *sir3Δ*, *sir2Δ*, and *sir1Δ* mutants is not due to the cell's diploid-like state.

### *HML*-*HMR* Interaction Does Not Require Nuclear Periphery Attachment

*HML* and *HMR*, as well as the telomeres, are clustered in silent compartments near the nuclear periphery [12],[13]. We questioned whether anchoring of these loci to the nuclear envelope (NE) may facilitate long-range interactions between them. To address this issue we wished to determine whether *HML* and *HMR* can interact and colocalize when their peripheral localization was disrupted. Two partially redundant pathways are involved in tethering heterochromatic loci such as telomeres to the NE. The first pathway is dependent on the Sir4p and Esc1p proteins. It has previously been shown in G1 cells that anchoring of the *HM* loci to the NE is reduced in cells deleted for Sir complex components [46],[47] and that Sir-dependent anchoring requires Esc1p. The second pathway requires the yKu70p/yKu80p heterodimer. If either one of these genes is deleted most telomeres are partially released from the periphery [16], [47]–[49], although *HM* loci remain peripherally located [47].

**Figure 4:**
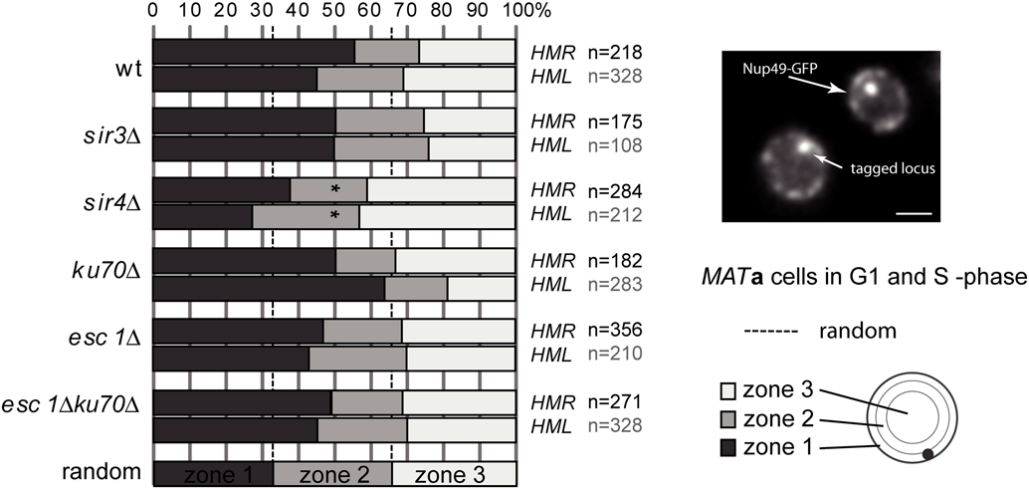
Preferred peripheral position of *HML* and *HMR*. Positions were mapped relative to the nuclear envelope in strains of GFP-tagged *HML* and *HMR* loci in WT, *sir3Δ*, *sir4Δ*, y*ku70Δ* and *esc1Δ* G1 and S phase *MAT* *a* cells. Data are represented in bar graphs as the percentage of spots in 3 concentric zones of equal surface. The number of cells analyzed (n) is indicated. * indicates a distribution identical to random (33% in each zone; *P*>0.05) in a 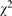 analysis. Bar is 1 µm.

Figure 4 shows the radial position of the *HM* loci for WT and mutant strains in interphase (and not only G1) cells to be directly comparable with 3C studies that involve analysis of non-synchronized cultures (see below). Nuclear positions of the *tet* ^op^ and *lac* ^op^ tagged silent *HML* and *HMR* loci were visualized using TetR or LacI repressor-GFP fusion proteins in G1 and S-phase cells [47],[50]. Data were acquired in three dimensions to assign the position of the resulting fluorescent spot relative to the GFP-tagged NE (Nup49-GFP) in the focal plane in which it was brightest and in one of three concentric nuclear zones of equal surface. Enrichment of the silent mating type loci near the nuclear envelope in wild-type cells is abolished in a *sir4*Δ strain [[47]; Figure 4]. Interestingly, we find that this effect is specific for *sir4Δ* cells: in *sir3*Δ mutants significant anchoring of both *HML* and *HMR* was retained, possibly due to binding of Sir4p to the silencer nucleation site (Figure 4). Since in *sir3Δ* mutant strains we no longer detected preferential interaction and colocalization of *HML* and *HMR* (Figure 3), we conclude that proximity to the NE is not sufficient for their interaction. In addition, we find that in *esc1Δ* cells, the *HM* loci maintain their peripheral localization. This suggests that Sir4p containing heterochromatin can associate with the nuclear periphery in an Esc1p-independent manner.

*HML* and *HMR* are also both directly associated with the nuclear periphery in a manner that does not require yKu70p. In interphase the position of *HMR* is unaffected by the absence of yKu70p, while *HML*'s anchoring to the NE is significantly increased ([47], Figure 4). Further, the increase in peripheral localization of the GFP-tagged *HML* locus in *yku70Δ* cells is dependent on the presence of the *HML* locus [47]. Similarly, deletion of *HMR* reduces the peripheral localization of the right end of chromosome III (KB, unpublished observations). These analyses suggest that peripheral localization of *HM* loci is not solely due to the close proximity of *HM* loci to telomeres that are often anchored to the nuclear periphery and further indicate that the *HM* loci strongly contribute to the peripheral localization of the ends of chromosome III.

Next we analyzed strains deleted for both *ESC1* and *YKU70* in which both anchoring pathways are abolished. We find that peripheral anchoring of *HML* and *HMR* was only slightly but not significantly reduced in interphase cells (Figure 4). Anchoring was somewhat more reduced in G1 than in S phase cells (data not shown). These observations on the radial position of *HMR* in its native chromosomal location extend those reported by Gartenberg and colleagues who found that deletion of both *YKU70* and *ESC1* resulted in loss of peripheral localization of an extrachromosomal *HMR* locus [46]. Our results suggest that chromosomal context plays a role in peripheral localization. We conclude that alternative Sir4p-dependent pathways exist that anchor *HM* loci to the nuclear periphery.

**Figure 5:**
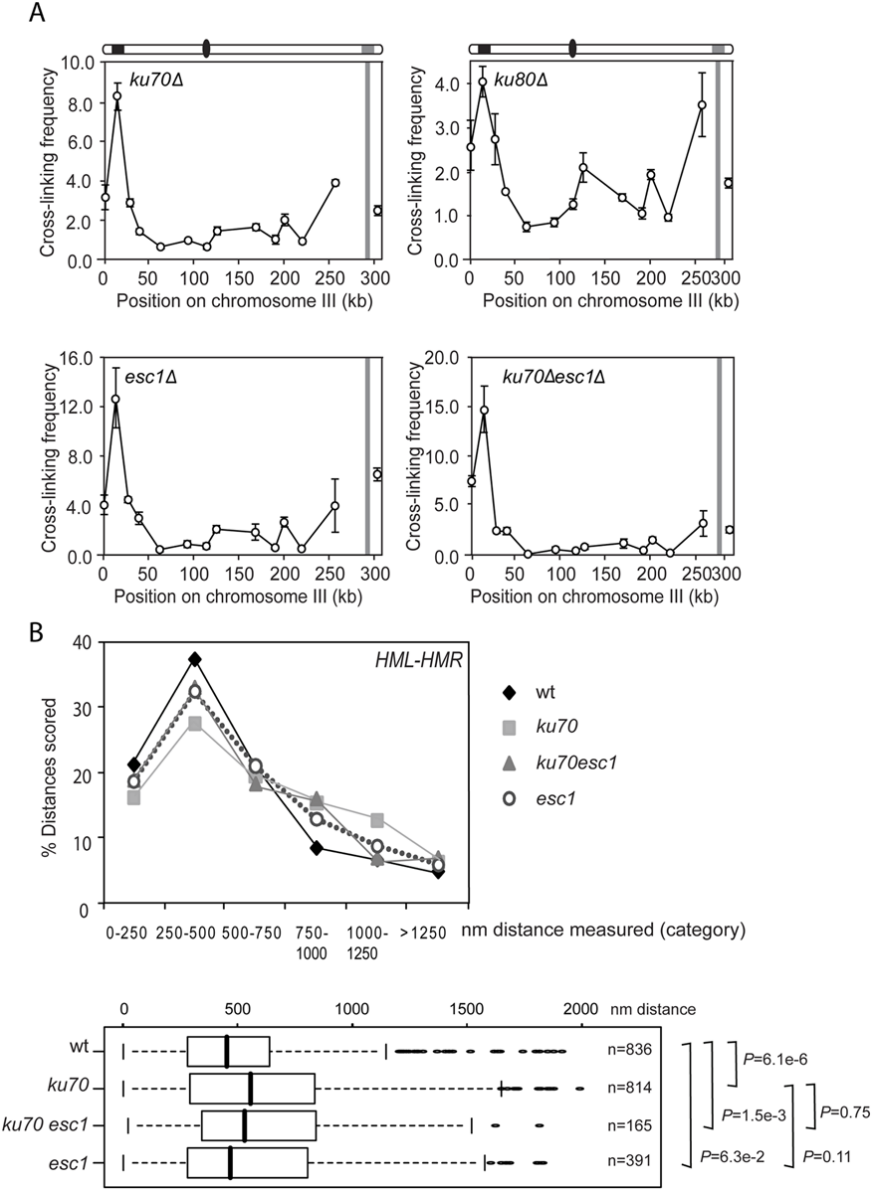
*HM* Interaction is independent of telomere anchorage mutants. (A) 3C analysis of interactions in *yku70Δ*, *yku80Δ*, *esc1Δ*, *and yku70Δesc1Δ* cells examining cross-linking frequencies between the *EcoRI* fragment containing *HMR* with other restriction fragments along the length of chromosome III. (B) 3D live cell analysis of *HML-HMR* colocalization in *yku70Δ*, *esc1Δ* and y*ku70Δesc1Δ* cells. Image stacks were acquired and analyzed as in Figure 1. Distance distributions of *HML* and *HMR* represented in box plots. Probabilities were calculated in R using the Wilcox test.

Next we analyzed *HM* interactions by 3C. In *yku70Δ* and in *esc1Δ* cells, we observed a significant increase in the frequency with which *HML* and *HMR* interact as compared to wild type cells (Figure 5A). Deletion of Y*KU80* did not significantly affect the crosslinking frequency. In y*ku70Δ esc1Δ* double mutants the *HML*–*HMR* crosslinking frequency is slightly higher than in either single mutant. These results demonstrate that yKu70p and Esc1p are not required for *HML* and *HMR* to specifically interact.

We also analyzed *HML*–*HMR* colocalization in these strains by live cell fluorescence microscopy (Figure 5B). In *esc1Δ* cells 51% of cells measured exhibit *HML* and *HMR* colocalization or juxtaposition (n = 391), which is comparable to what we observed in wild type cells (*P* = 0.063 wt vs *esc1*). In *yku70*Δ cells *HML* and *HMR* are found colocalized in 18% of the cells and adjacent to each other in another 28% of cells (n = 814). The frequency of co-localization is comparable to wild type, but the distribution of distances between *HML* and *HMR* differs from wild type. It appears that in the absence of yKu70p more nuclei display widely separated loci than in wild type (*P* = 6.1e^−6^ wt vs *yku70Δ*). This may be related to the fact that populations of *yku70Δ* cells display two semi-stable states of silencing: one in which telomeres are delocalized and one in which they remain clustered [51]. Finally, we found that colocalization of *HML* and *HMR* in *yku70Δesc1Δ* double mutants was also comparable to wild type: 52% of the cells measured still show *HML* and *HMR* colocalized or immediately adjacent to each other (n = 165) (Figure 5B).

We note that the 3C analysis revealed a ∼4-fold increase in *HML*-*HMR* interactions in all three mutants, but that live cell fluorescence failed to detect an increase in colocalization of these loci. The increased crosslinking frequency as detected by 3C may be due to loss of interactions of *HM* loci with telomeres, which could result in an increased chance for *HM* loci to become ligated in the 3C assay or due to a more intimate association that is more easily crosslinked (see discussion).

**Figure 6:**
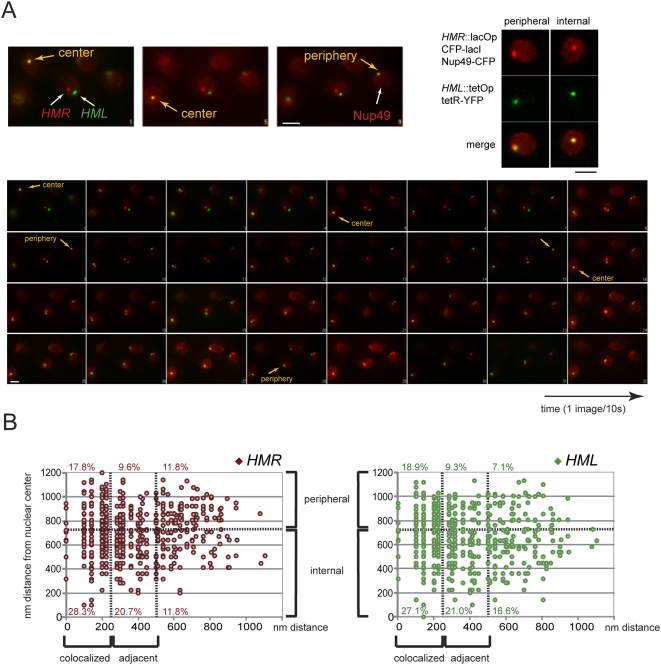
Interaction between *HML* and *HMR* is independent of peripheral anchoring. (A) Time lapse images of YFP and CFP tagged *HML* and *HMR* relative to the nuclear rim as identified by Nup49-CFP. Images were acquired on a wide-field Olympus XI microscope using a 100× objective (NA = 1.4) every 10 s for 320 s without changing the focal plane. Acquisition times were 400 ms for each wavelength. *HML* and *HMR* colocalize transiently near the periphery or in the center of selected nuclei (yellow spots). Bar is 1 µm. (B) Position of *HML* and *HMR* relative to the nuclear center inferred from tetR-YFP nuclear fluorescence in single nuclei over time taking images in 2D every 1.5 seconds on a confocal LSM510 microscope plotted against the distance between *HML* and *HMR* at every time point during 1–3 min time lapse movies. Colocalization was assigned for distances <250 nm, juxtaposition for distances <500 nm (adjacent). The interior fraction corresponds to a position of *HML* or *HMR* at less than 720 nm from the nuclear center, thus about 2/3 of the positions. The percentages represent the fraction of datapoints in any sector of the graph.

These analyses show that *HML*-*HMR* interactions do not require the known membrane anchors yKu70p/yKu80p and Esc1p. However, their peripheral localization was also not abolished in the absence of both these membrane anchors. Our inability to genetically disrupt peripheral localization of the silent mating type loci prevented us from directly assessing the influence of membrane anchoring on facilitating *HML*-*HMR* interactions. Therefore, as an alternative approach, we set out to follow the positions of *HML* and *HMR* in wild type living cells over several minutes in order to determine whether *HML-HMR* interactions can be observed in the interior of the nucleus or only at the periphery (Figure 6). Figure 6A shows a representative movie comprising a series of 50 2D images taken at 10 s intervals of interphase cells on a wide-field Olympus XI inverted microscope. *HML* and *HMR* tags colocalized either near the NE as identified by the nuclear pore component Nup49p fused to CFP, or at the center of the nuclei imaged. In addition, colocalization was a transient event, because, after a few minutes, separation of previously colocalized loci was observed (compare time points 9 and 12 or time points 4 and 5). Thus, *HML* and *HMR* seem to collide and separate both at peripheral and internal nuclear locations. In order to determine whether *HML* and *HMR* were more likely to interact at the nuclear periphery, we followed their position relative to the nuclear center in single nuclei over time taking images in 2D every 1.5 seconds on a confocal LSM510 microscope. Figure 6B summarizes the distances between *HML* and *HMR* plotted against the distance of either *HML* or *HMR* to the nuclear center at every time point during nine 1–2 min time lapse movies (n = 676). We found that in 2D *HML* is separated <250 nm from *HMR* (colocalization) in >45% of the time points scored. Moreover, the probability of colocalization was similar in the interior fraction (position of *HML* or *HMR* less than 720 nm from the nuclear center, about 2/3 of the positions) and the peripheral fraction of the nucleus. These movies clearly demonstrate that over long periods, interaction between *HML* and *HMR* was independent of NE anchoring.

### *HML* and *HMR* Interaction Is Dependent on Two Pathways for Nucleosome Assembly

Histone chaperones and other proteins involved in nucleosome assembly play roles in gene silencing, heterochromatin formation and heterochromatic clustering in a number of organisms including yeast [26], [27], [52]–[55]. We determined whether these activities are also required for interactions between *HML* and *HMR*. Yeast contains two histone assembly complexes. The chromatin assembly factor 1 (CAF-1) complex is involved in nucleosome assembly in S phase [56], whereas the HIR complex functions primarily outside of S phase [57]. The histone chaperone Asf1p stimulates the activity of both complexes [27],[58]. In addition, the HIR and CAF-1 complexes are involved in two parallel, and partially redundant pathways that enhance silencing at *HML* and *HMR* [27],[59].

**Figure 7:**
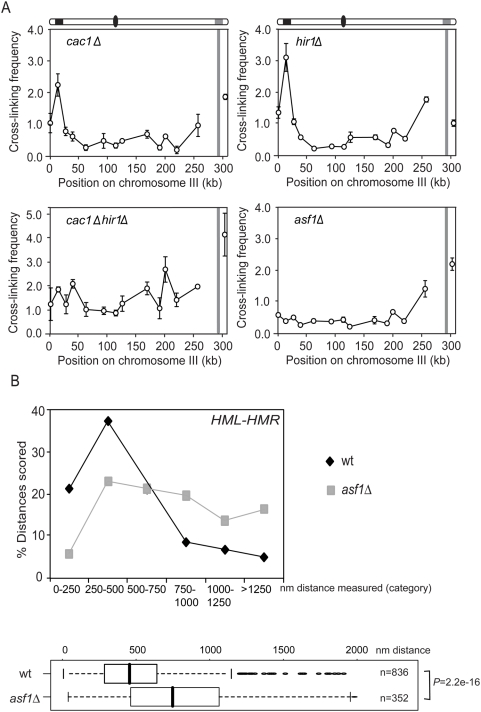
Role of nucleosome assembly factors in *HM* interactions. (A) Analysis of interactions in *cac1Δ*, *hir1Δ*, *cac1Δhir1Δ*, and *asf1Δ* cells examining crosslinking frequencies between the *Eco*RI fragment containing *HMR* with other restriction fragments along the length of chromosome III. (B) 3D live cell analysis of *HML-HMR* colocalization in *asf1Δ* cells. Image stacks were acquired and analyzed as in Figure 1. Distance distributions of various telomere pairs represented in box plots. Probabilities were calculated in R using the Wilcox test.

First, we tested a strain in which the CAF-1 complex is disrupted by deletion of *CAC1*. Cac1p is the largest subunit of the CAF-1 complex and in strains lacking Cac1p *HML* and *HMR* are weakly derepressed [26]. We find that deletion of *CAC1* did not affect the frequency with which *HMR* and *HML* interact (Figure 7A). Next we analyzed a strain lacking Hir1p, a subunit of the HIR protein complex. Deletion of *HIR1* has also been reported to result in slight de-repression of *HML* and *HMR* [57]. As for *cac1Δ* strains we find that deletion of *HIR1* has no effect on the frequency of the *HML*-*HMR* interaction (Figure 7A). Given the known functional redundancy of HIR and CAF-1 complexes, we created a double mutant strain in which both *CAC1* and *HIR1* are deleted. We find that in this case the prominent interaction between *HML* and *HMR* is no longer observed (Figure 7A). These results point to a role of nucleosome assembly in mediating *HML-HMR* interactions, and show that the HIR complex and CAF-1 complex are functionally redundant in this process. Interestingly, in the double mutant, interaction frequencies along the entire chromosome are two- to three-fold higher than the background interactions we observed for all other strains. This may point to a more flexible chromosome organization.

To further investigate the role of nucleosome assembly, we studied a strain lacking the histone chaperone Asf1p which functions with both the CAF-1 and HIR complex. Recently a role for Asf1p has been proposed for telomere sub-nuclear positioning [60]. Interestingly, we find that in an *asf1Δ* mutant the interaction between *HML* and *HMR* is no longer observed (Figure 7A). We have also analyzed *asf1Δ* cells by live cell fluorescence microscopy (Figure 7B). As compared to wild-type where in ∼60% of the cells *HML* and *HMR* are found colocalized or adjacent to each other, *asf1Δ* cells only have 29% of cells with *HML* and *HMR* colocalized or adjacent to each other (n = 352; *P* = 2.2e^−16^ wt versus *asf1*).

Asf1p is also required for Histone H3K56 acetylation and its deposition [61],[62]. H3K56 acetylation and deacetylation has been found to play roles in silencing telomeric loci in yeast and tethering of telomere 14L [60],[63]. For this reason we chose to analyze strains which lack Rtt109p, the enzyme that acetylates Histone H3K56 in an Asf1p-dependent manner [62],[64],[65]. We find that in this mutant the interaction between *HML* and *HMR* is still observed, albeit with a somewhat reduced crosslinking frequency as compared to wild-type. (Figure S3B). Therefore, the loss of interaction observed in *asf1Δ* mutants is not solely due to a loss of Histone H3K56 acetylation. Thus, incorrect or unstable tetramer incorporation may lead to poorly organized chromatin that is unfavorable for heterochromatic loci to interact.

### Relationship between Silencing and *HM* Interactions

**Figure 8:**
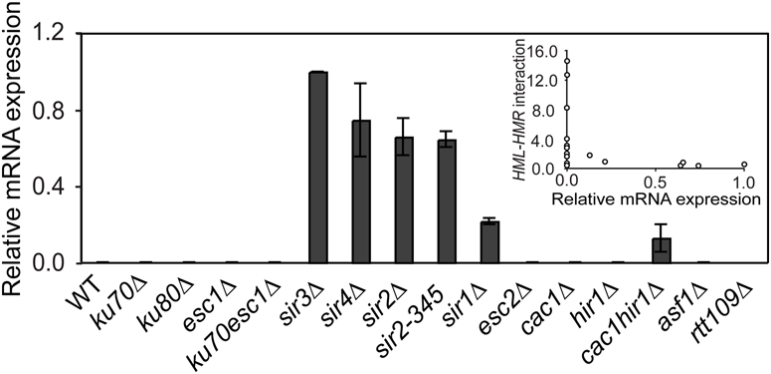
Silencing and *HM* interactions are distinct processes. RT-PCR analysis of *a* *1* transcription in wild-type and mutant *MATα* strains analyzed by 3C. Error bar represents standard error of the mean from two independent experiments. Expression of *ADH1* was used as an internal control and data was normalized to level of expression in *sir3Δ* mutants (set at 1). Inset: Crosslinking frequency of *HML-HMR* interaction is plotted versus the relative mRNA expression of *a* *1* for all mutants analyzed to indicate that no clear correlation between silencing and *HM* interactions is detected.

In order to determine whether *HML*-*HMR* crosslinking frequencies and colocalization are related to silencing, we measured the level of silencing in the various mutant strains analyzed in this study. Previously, varying silencing defects were observed for the strains described here that display loss of the *HML*-*HMR* interactions (*sir1Δ*, *esc2Δ*, *asf1Δ* and *cac1Δhir1Δ*) [27],[42],[43]. In most cases silencing defects could be detected using strains that have a reporter gene inserted in one of the *HM* loci (either *URA3* or *ADE2*). Use of a reporter provides highly sensitive assays to quantify de-repression of *HM* loci as expression of the reporter gene can be detected even when expressed at very low levels. However, these reporter assays do not quantify the level of mRNA production compared to a fully expressed or repressed state. In order to quantify mRNA levels of the endogenous genes directly in a population of cells we employed RT-PCR to quantify the level of ***a*** *1* mRNA levels (located at *HMR*) in *MATα* strains. This allowed us to analyze silencing levels in the same strain in which interactions between *HM* loci were detected. We observe that deletion of *SIR4*, *SIR3*, *SIR2*, *SIR1* or combined deletion of *CAC1* and *HIR1* as well as a *sir2-345* point mutation results in significant de-repression of *HMR* relative to the *Adh1* gene that was used as a normalization control (Figure 8). However, the other two mutants that display loss of the *HM*-interactions (*esc2Δ* and *asf1Δ*) had no detectable levels of **a** *1* expression, and thus had largely normal levels of silencing as determined by RT-PCR. Experiments employing reporter genes also revealed only very minor silencing defects in these strains [43],[66].

We conclude that all mutants with a significant silencing defect have also lost the interaction between the *HM* loci. However, we find no clear quantitative correlation between silencing and *HM* interactions (Figure 8, inset) because in two cases (*esc2Δ* and *asf1Δ*) *HM* interactions are lost, but silencing is mostly unaffected. We conclude that Sir-mediated silencing is not sufficient for clustering of *HM* loci, and that additional processes in heterochromatin formation are specifically required for associations between heterochromatic loci. These processes require at least Asf1p, Esc2p and possibly Sir1p.

## Discussion

Here we report specific long-range interaction between the two silent *HM* loci that is most prominent around their flanking regions encompassing the *HML-E* and *HMR-I* silencer elements. This interaction was carefully documented using 3C and 3D live cell imaging technologies in parallel. Previously, we performed 3C on purified nuclei and also found relatively frequent interactions between the ends of chromosome III, including *HML* and *HMR* [29]. However, in that analysis the interaction between these loci was significantly lower compared to the data presented here and the *HML*-*HMR* interaction was not more frequent than the interaction between the two telomeres. Thus, it appears that the *HML*-*HMR* interaction is specifically lost during the nuclear isolation procedure, highlighting the importance of analyzing intact cells.

The interaction between *HML* and *HMR* requires Sir1p, Sir2p, Sir3p, Sir4p and Esc2p as well as nucleosome assembly complexes, known to be involved in establishment and maintenance of heterochromatin at these loci. Our analysis reveals that *HM* loci can be silenced without clustering and suggests that gene silencing and clustering are distinct properties of heterochromatin. We propose that interaction of heterochromatic domains requires proper chromatin conformation that depends on at least Asf1, Sir1p and Esc2p.

### Membrane Association and Clustering of *HM* Loci Are Independent

Our live tracking of the sub-nuclear positions of *HML* and *HMR* in wild-type cells showed that these loci can colocalize as frequently when these loci are located near the center of the nucleus as when they are near its periphery. Therefore, association with the nuclear envelope is not required for *HML*-*HMR* interactions. Further, the results we obtained in a *sir3Δ* strain show that membrane anchoring is not sufficient for *HML*-*HMR* interactions to occur. We conclude that clustering of heterochromatic loci and NE anchoring are two distinct processes that each contribute to nuclear organization.

Surprisingly, our analyses also reveal that peripheral localization of *HM* loci is not reduced upon inactivation of the two previously defined pathways for membrane anchoring of heterochromatin. As shown previously [47], deletion of *YKU70* increases the peripheral localization of *HML* in interphase cells, while not affecting the positioning of *HMR*. In addition, given that in *yku70Δesc1Δ* double mutant cells heterochromatin formation near telomeres is disrupted and Sir4p is found uniformly throughout the nucleus [46],[48] but *HM* interactions are retained (Figure 5), these data also indicate that sequestration of Sir protein in clusters is not required for interactions between *HML* and *HMR*. Consistent with the loss of Sir protein clusters, we found that in *yku70Δesc1Δ* double mutant cells interactions between some telomeres is reduced, as detected by 3C, suggesting that in this mutant telomere clustering is affected, while *HM* interactions are retained (Figure S3D).

We observed that the interaction between *HML* and *HMR*, as detected by 3C, is more frequent in *yku70Δ*, *esc1Δ*, *and yku70Δesc1Δ* mutant cells than in wild-type cells. However, live fluorescence microscopy revealed no increase in their colocalization as compared to wild type cells. This apparent discrepancy may be explained in different ways. First, it is possible that the interaction between *HML* and *HMR* is more intimate, and thus more effectively crosslinked, in these mutants, in a manner that is not microscopically distinguishable from wild type. A more intimate association may be the result of an increase in Sir protein occupancy at the *HM* loci in these mutants. Sir protein occupancy at the *HM* loci is likely increased in these mutants because telomere silencing and clustering are affected [46],[49]. Loss of Sir proteins from telomeres has been found to increase the available pool of Sir proteins that are accessible to the *HM* loci, which may enhance their ability to interact [14],[51]. An alternative explanation is that 3C underestimates the *HM* crosslinking frequency in wild type cells, or overestimates their crosslinking frequency in these mutants. Given that the *HM* loci interact with each other as well as with telomeres, a loss of telomeric heterochromatin will reduce the number of interaction partners to which *HM* loci can be cross-linked and ligated during the 3C procedure, resulting in detection of relatively more frequent *HML*-*HMR* interactions in these mutants. Although our studies clearly found a strong correlation between 3C crosslinking frequency data and colocalization of *HM* loci, future experiments are needed to further quantify the relationship between 3C data and data obtained by fluorescence microscopy.

### *HM* Interactions and Silencing Are Related but Mechanistically Distinct Processes

The Sir complex and proteins that recruit this complex, as well as histones and nucleosome assembly factors all cooperate to assemble silenced chromatin domains. We find that mutants in each of these protein complexes display loss of *HML*-*HMR* interactions. Thus the pathways that mediate heterochromatin formation and the mechanism(s) that drive *HM* interactions are clearly related and are mediated by overlapping protein complexes. However, several observations indicate that these processes are mechanistically distinct as they differentially depend on specific chromatin factors.

First, two mutants (*esc2Δ* and *asf1Δ*) that display a complete loss of interaction between *HML* and *HMR* display only very minor defects in silencing as detected by RT-PCR. Similar very minor silencing defects have been detected using reporter constructs. Huang et al. used a GFP reporter gene inserted in *HMR* to detect GFP expression in WT, *sir3Δ* and *asf1Δ* strains [66]. They found that in WT cells *HMR* is silent and only 0.3% of cells expressed GFP, whereas in *sir3Δ* cells the locus was mostly derepressed with 99% of cells expressing GFP. Deletion of *ASF1* resulted in GFP expression in only 0.9% of cells, indicative of effective silencing. Although we cannot formally exclude the possibility that very small defects in silencing are sufficient to cause loss of *HML-HMR* interactions, we favor the interpretation that heterochromatin formation and silencing is not sufficient for *HM* interactions, and conversely that *HM* interactions are not essential for heterochromatin formation.

Second, genetic evidence indicates that Hir1p and Asf1p act in the same silencing pathway [27], but they display very different effects on *HM* interactions, suggesting that for the latter process they act in different pathways. In addition, single deletions of *HIR1*, *CAC1* or *ASF1* all result in minor silencing defects [26],[27],[66],[67], but only deletion of *ASF1* results in loss of *HM* interactions. Third, the *cac1Δhir1Δ* double mutant and the *asf1Δ* mutant display the same loss of *HML*-*HMR* interactions, but they have quantitatively very different effects on silencing [27], again pointing to differential dependence of silencing and long-range interactions on these chromatin assembly factors.

The differential effect of deletion of *SIR1* on silencing and *HM* interactions is particularly interesting. In the absence of Sir1p *HML* and *HMR* are partially derepressed. This is due to the occurrence of two distinct populations of chromatin states: in one subset of cells the loci are completely repressed, whereas in the other subset they are fully expressed [42]. Our RT-PCR analysis of *HMR* expression suggests that ∼60% of loci remain repressed, whereas ∼40% are expressed. However, the 3C analysis showed a complete loss of *HML*-*HMR* interactions and the level of colocalization, as determined by live cell 3D imaging was indistinguishable from background levels observed in *sir4Δ* cells. These results indicate that Sir1p may have a specific role in long-range interactions between heterochromatic loci that is distinct from mediating gene silencing per se.

Taken together, formation of silent heterochromatin at *HML* and *HMR* is essential but not sufficient for long-range interactions between *HM* loci. *HML*-*HMR* interactions require Asf1p, Esc2p and possibly Sir1p in a process that is distinct from silencing. Other proteins, such as other SIR complex components may also play a role in that process, but their role in *HM* interactions is more difficult to assess, as they are also essential for *HM* silencing.

Interestingly, it has recently been shown that deletion of *ASF1* shows a telomere-positioning defect, and also affects the sub-nuclear positioning of other chromosomal loci [60]. Deletion of *ASF1* does not affect telomere silencing [27],[67], suggesting that silencing of telomeres is not sufficient for their positioning. Combined with our data, these results point to a specific Asf1p – dependent process that is required for heterochromatic positioning in the nucleus in general.

### Potential Role of Silencer Elements in Long-Range Interactions

Restriction fragments encompassing *HML-E* and *HMR-I* interact most frequently compared to other parts of the *HM* loci. These restriction fragments do not contain the boundary elements that have been identified up and downstream of *HMR* [68], suggesting the *HM* interaction involves the *HML-E* and *HMR-I* silencer elements specifically. Interestingly, one protein that we identified as critical for long-range interactions, Sir1p, associates with replication related complexes bound to the silencer elements [69],[70], and another (Asf1p) displays genetic interactions with ORC2 [71] and physically associates with other replication factors such RF-C [72]. Unfortunately, we have not been able to precisely delete *HMR-I* so we have not been able to directly test the role of this element in mediating *HM* interactions. Deletion of *HML-E* would not address this issue as it would also result in derepression of *HML*.

Our results are different from those recently described by Kamakaka and co-workers, who reported Sir3p-dependent looping interactions between the two silencers of *HMR*, *HMR-E* and *HMR-I* [28]. One explanation for this difference could be the fact that they used strains in which *HMR* was slightly modified to introduce extra *Sau*3A restriction sites, and *HML* and the active *MAT* locus were deleted, precluding *HMR*-*HML* interactions. However, we did not detect *HML-E*-*HML-I* interactions when we deleted *HMR* in our strain background (not shown). An alternative explanation is that different strain backgrounds were used.

### Clustering of Heterochromatic Loci and Nuclear Compartmentalization

The *HML*-*HMR* association is not essential for silencing, suggesting that the role of this interaction in silencing is either highly redundant with other pathways that contribute to heterochromatin formation or that it is involved in other processes, such as mating type switching or contributing to higher order nuclear organization in general.

Various lines of evidence suggest that *HM*-interactions are not involved in mating type switching. First, we did not detect any differences in the interaction in either *MAT* ***a*** or *MATα* cells, despite well-characterized mating-type-dependent differences between the left and right arm of chromosome III with regards to mobility and accessibility for recombination complexes [e.g. [73],[74]]. Second, we also analyzed mutants in which the recombination enhancer (RE), an element known to mediate donor preference in mating type switching, was deleted and observed only a minor increase in crosslinking frequencies between *HML* and *HMR in* ***a***- or α- cells (Figure S2C and D). In addition, previous studies also revealed no difference in nuclear positioning of *HML* and *HMR* in mutants when the RE was deleted [47] (and data not shown). Lastly, we tested directly whether any mating type switching defect was observable in *asf1Δ* and *esc2Δ* mutants. We analyzed the mating proficiency of meiotic products of *HO/” asf1Δ/”* or *HO/” esc2Δ/”* strains and found that spore colonies did not mate with a tester strain, indicating that spores efficiently self-diploidized and thus were fully capable of switching (data not shown). Thus, it appears that the *HM*-interaction does not play a critical role in mating type switching.

**Figure 9:**
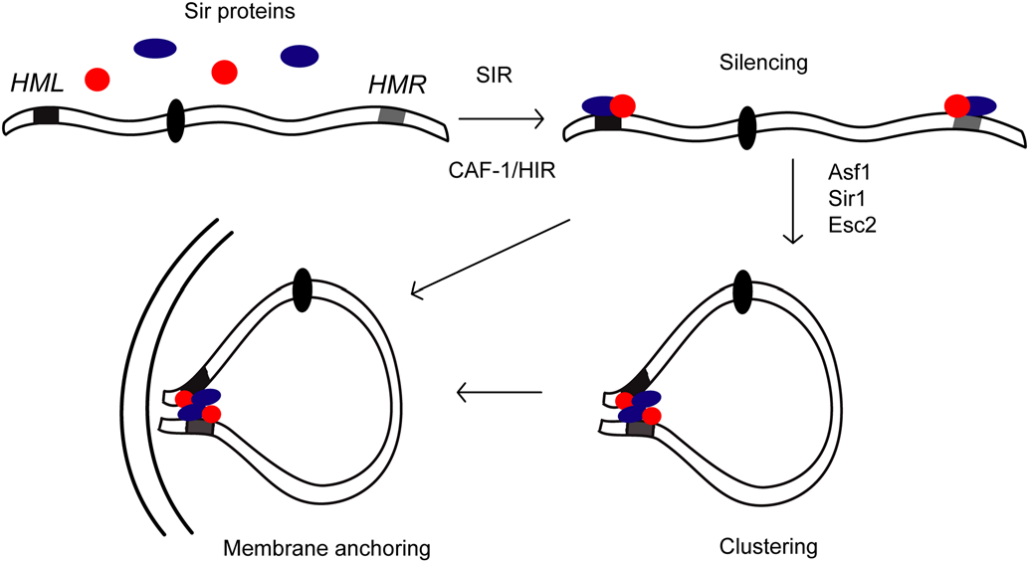
Clustering of silent chromatin. In the absence of silencing proteins, depicted as colored circles, *HM*-loci (*HML*- black rectangle; *HMR*- gray rectangle) are not silenced and they do not form clusters. Upon expression, silencing proteins are available throughout the nucleus and can become recruited to the *HM* silencers, independent of their sub-nuclear position. Heterochromatin is formed and silencing is established. Once these loci are silenced, they obtain the ability to form clusters that can also contain telomeres. Silent clusters create sub-nuclear compartments in which there is a high local concentration of silencing proteins available for recruitment to loci located within the cluster only. Membrane association of silenced loci will anchor clusters or unclustered loci to the nuclear periphery. Loci located outside heterochromatin compartments have no access to silencing proteins and are not silenced. In this model clustering, membrane anchoring as well a genomic proximity of *HM* loci to telomeres will all contribute to formation of sub-nuclear compartments that are enriched in silencing complexes, which will facilitate heterochromatin formation at resident loci.

We propose that the *HML*-*HMR* interaction plays primarily a structural role by contributing to clustering of heterochromatin and formation of heterochromatic sub-nuclear compartments (see Figure 9 for model). In the absence of silencing proteins, *HM*-loci are not silenced and do not interact. Upon expression of silencing proteins, they are recruited to the *HM*-silencers resulting in heterochromatin formation and silencing. This step requires the presence of SIR proteins and the CAF-1/HIR1 complex for proper nucleosome assembly. Once silenced, these loci then engage in long-range interactions, which are dependent upon the presence of Asf1p, Esc2p, and Sir1p. Once heterochromatic clusters are formed, the nuclear distribution of silencer proteins becomes highly non-homogeneous with high local concentrations in the silent compartments and depletion in the rest of the nucleus. Nuclear compartmentalization could be advantageous because only loci located within the silent compartments will have access to abundant silencer proteins while the rest of the genome is precluded from inadvertently gaining access to heterochromatin proteins. Consistent with this model, a recent study showed that loss of heterochromatic clustering resulted in inappropriate Sir-mediated ectopic repression of genes throughout the genome [75].

## Materials and Methods

### Yeast Strains Construction and Growth

*HMR*, *SIR1*, *SIR2*, *SIR3*, *SIR4*, *CAC1*, *ASF1*, *ESC2*, *KU70*, *KU80*, *ESC1*, *HIR1*, the recombination enhancer (RE) located on chromosome III (SGD coordinates 29017–29799) and *RTT109* were replaced with antibiotic resistance markers using a standard PCR based gene disruption strategy [76]. To generate the *sir2-345* mutant, plasmid pRS 345 [23], which is linked to *LEU2* was cut with Hpa1 and integrated into chromosome III in a *sir2Δ* background. All strains used for 3C were maintained in a SK1 background (Supplementary Table 2). Cells were freshly streaked on YPD medium containing 2% glucose before 3C analysis. For microscopy analyses, yeast were grown at 30°C in rich glucose media (YPD) unless otherwise indicated. Strains are listed in Table S2. Plasmids used to integrate the tet or lac operator arrays and repressors were as described [77]. The following PCR-amplified genomic fragments (SGD coordinates) were used for insertion near the respective loci: 15160–15773 for *HML*, 294892–295241 for *HMR* and 197194–196910 for *MAT* on Chr3. LacI-GFP or lacI-CFP, and tetR-GFP or tetR-YFP, and where indicated GFP-Nup49 or CFP-Nup49 fusions, were introduced as described [77]–[79]. Note that the wild type strain with the *tet* ^op^ and *lac* ^op^ operator inserted near the *HM* loci has been described before [30].

### Image Acquisition and Manipulation

For live and intact cell imaging, cultures carrying two color tagged integration sites were grown exponentially in YPD to OD_600 nm_< = 0.4 (∼1×10^7^ cells/ml), and rinsed in complete synthetic medium before imaging. Microscopy was performed at 25°C. Cells were spread on synthetic complete 3% agarose patches for acquisition.

3D images were captured on a Metamorph driven Olympus IX81 wide-field microscope equipped with a Coolsnap HQ camera and a Polychrome V (Till Photonics). Stacks of 21 images were acquired with a step size of 0.2 µm either at 490 nm for 200 ms for GFP or alternating the wavelength between 435 nm (CFP) and 512 nm (YFP) at every image plane, and exposures of 400 ms per wavelength. A 100×/1.4 Oil Plan-Apochromat objective (Olympus) was used. Position of GFP tagged loci relative to the nuclear rim identified by the Nup49-GFP signal were determined as a percentage of fluorescent spots in one of three concentric nuclear zones of equal surface in the plane bearing the brightest GFP-lacI or tetR-GFP focus using the Pointpicker plug-in for Image J [47],[50]. Only the 10 core focal planes were scored. For 3D distance determination, CFP and YFP images were automatically analyzed using SpotDistance implemented as a plug-in for ImageJ, freely available at: http://bigwww.epfl.ch/spotdistance/ [80]. The measured distances were loaded into R software (www.r-project.org) and the measured distance distributions translated into a box plot. Outliers are defined as 1.5 times the Inter Quartile Range (IQR) and are represented as open circles. Different distance distributions (determined as non-normal using a Kolmogorov-Smirnov test) were scored for significant difference in R using the Wilcox test.

2D time-lapse imaging (Figure 6B) was performed on a Zeiss LSM510 confocal microscope using a 100× Plan-Apochromat objective (NA = 1.4) partly at the RIO Imaging facility in Toulouse, France, and partly in Susan Gasser's laboratory at the University of Geneva, Switzerland. Live imaging was performed as described [30]. Nine independent 2D time-lapse series of 50 to 150 confocal images were acquired at 1.5 s intervals of G1-phase nuclei, following the tagged foci by adjusting the focal plane. 2D distances were measured manually using Metamorph.

### 3C Analysis

3C was performed as described by Dekker et al., with modifications described in Miele *et al.* [4],[29],[32]. Cells were freshly grown in YPD medium to an OD_600_ of 1.0. Cells were then resuspended in buffer containing 0.4 M sorbitol, 0.4 M KCl, 40 mM Na(H)PO_4_, 0.5 mM MgCl_2_, and 0.1 mg/mL of zymolyase 100-T and incubated at 30°C for 40 minutes. Efficiency of the digestion of cell wall was tested by observation of hypotonic lysis within 1–2 minutes. Spheroplasts were washed three times in a buffer containing 0.1 M MES, 1.2 M sorbitol, 1 mM EDTA, and 0.5 mM MgCl2 and then resuspended in the same buffer. Formaldehyde was added to a final concentration of 1% and crosslinking was allowed to proceed for ten minutes at room temperature. The reaction was quenched by addition of glycine to a final concentration of 125 mM and incubation for 5 minutes at room temperature. Cells were washed three times and resuspended in the restriction enzyme buffer. Chromatin was solubilized by the addition of 0.1% final concentration of SDS and incubation for 10 minutes at 65°C. Trition X-100 was added to a final concentration of 1%. A restriction enzyme was added and samples were incubated overnight at the appropriate incubation temperature. The digestion efficiency was determined to be 80% and this percentage did not vary between restriction cut sites (see Figure S3C). The restriction enzyme was inactivated by addition of SDS to a final concentration of 1.6% followed by incubation at 65°C for 20 minutes. Chromatin was diluted for ligation to promote intramolecular ligation over intermolecular ligation. Triton X-100 was added to 1% and DNA was ligated for 2 hours at 16°C with T4 DNA ligase. The cross-links are reversed overnight by incubation at 65°C in the presence of 5 microgram/ml of proteinase K. After overnight incubation an additional 5 microgram/ml of proteinase K was added and incubated for an additional 2 hours at 42°C. DNA was purified by a series of phenol-chloroform extractions followed by ethanol precipitation. The resulting template was then treated with RNAse A.

In addition to the above 3C template a randomized control template was also generated which is used to determine PCR amplification efficiency of specific 3C ligation products. This template was created by digesting purified non-crosslinked yeast genomic DNA which is then followed by a random intermolecular ligation. The resulting template was purified by a series of phenol-chloroform extractions and ethanol precipitations. The resulting template was also treated with RNAse A.

Once both the 3C templates and control templates are generated a PCR titration is performed to determine the amount of template to be used in the subsequent PCR reactions as shown in Figure S1A and B. PCR is performed in a 50 µl reaction in a buffer containing 10 mM Tris-Cl pH 8.4, 50 mM KCl, 2.25 mM MgCl_2_, 0.5 mM dNTPs and 0.4 µM of each primer. The following PCR program gives quantitative results: 32 times: 1 minute at 95°C, 45 seconds at 60°C, 2 minutes at 72°C. This is followed by 1 minute at 95°C, 45 seconds at 60°C, and 8 minutes at 72°C. PCR products are quantified semi-quantitatively on 1.5% agarose gels in the presence of ethidium bromide. Previous work displays that semi-quantitative PCR and quantitative real-time PCR yield similar results [39],[81],[82]. Multiple primer combinations are used that detect highly abundant 3C ligation products as well as infrequently formed products to be sure that the amount of template used in each reaction is within the linear range. The subsequent PCR reactions are done in triplicate for both the control and 3C template. The crosslinking frequency is then determined by calculating the ratio between the amount of PCR product obtained with the 3C template and the control template. The three resulting crosslinking frequencies are then averaged together and one value is obtained. This is the value that is plotted in interaction graphs. The standard deviation of the mean is determined and is used as the error bars (see Table S3 for examples of crosslinking frequency determination).

Restriction enzymes were chosen to create an equal distribution of restriction fragment cut sites and also to allow for important elements (i.e. *HMR-I*, *HMR-E*, *HML-E*, *HML-I*, etc) to be on different restriction fragments. Primers were then designed for restriction fragments of interest (see Table S1 for sequences and Figure S1C for diagram of *EcoRI* primers). Primers were designed unidirectionally unless a unique primer could not be designed on that particular end of the fragment. They were designed to be approximately 28 base pairs long with 50% GC content. Once designed, the primers were tested on a control template and those primers that give aberrant products, multiple products, and abundant primer dimers were re-designed.

3C analyses for wild type and each mutant described above were performed in at least two, and in most cases three independent experiments, with each data point quantified in triplicate. Figure S4 displays some examples of biological repeats of 3C analyses that illustrate the highly reproducible nature of 3C interaction profiles in WT and several mutant strains.

All 3C data are normalized to the wild type (mating type **a**) dataset so that all interactions can be quantitatively compared, as described in [33],[34]. Datasets for wild-type (mating type α), *hmrΔ* fine mapping analyses, *ku70Δesc1Δ*, and *cac1Δhir1Δ* mutants strains were normalized by calculating the average log ratio of crosslinking frequencies between the anchor fragment and neighboring fragments measured within each of the different strains as compared to wild type and setting this ratio at zero. For data normalization with all other mutants, 8 crosslinking frequencies were determined between pairs of loci located along yeast chromosome VI. Data normalization was performed by calculating the average log ratio of the set of 8 crosslinking frequencies measured in each mutant as compared to wild type and adjusting the mutant data sets so that this ratio becomes zero.

### Digestion Efficiency Determination

*Eco*RI cutting efficiency was determined for a fragment upstream of *HML*, the fragment containing *HML*, and a fragment at the left telomere of chromosome III. Intact yeast cells were treated with 1% formaldehyde. Chromatin was solubilized and one part was digested with *Eco*RI while the other part was not. Cross-links were reversed and the DNA was purified. Semi-quantitative PCR was done across restriction fragments on both samples and the ratio of PCR products was calculated to determine the percentage of loci that were not digested upon formaldehyde cross-linking. The data was normalized for the amount of DNA used in each reaction using a pair of primers that amplify a region contained within an *Eco*RI fragment. Digestion efficiency was comparable to that reported previously [33],[34]. The level of digestion was around 75–80%, and was not significantly different for *HML* as compared to other sites (Figure S3C). Given that the digestion efficiency is linearly related to the level of cross-linking [34], this result also implies that the level of cross-linking is similar at these locations. We conclude that the peak in crosslinking frequency observed at *HML* and *HMR* is not due to differences in digestion or cross-linking.

### RT-PCR

Total RNA was isolated by using the RNeasy Mini Kit (Qiagen, Valencia, CA). cDNA was synthesized using the SuperScript First-Strand Synthesis for RT-PCR protocol including DNase I treatment as described (Invitrogen.com). The cDNA was synthesized using oligodT12-18 (Invitrogen) and then was amplified by PCR. Primer sequences for ***a*** *1* expression are described by Smeal *et al.* [83]. Primer sequences for *ADH1*, which was used as the internal control, are available upon request.

## Supporting Information

Figure S1 (A) The left (control template) and (B) right (3C template) panels represent titrations using four primer combinations: a close interaction (primer pair O16/O17; 10.1 kb- open circle), interaction between *HML* and *HMR* (primer pair O4/O22; filled circle), interaction between *HMR* and an element downstream of *HML* (primer pair O7/O22; black square); and an interaction between *HMR* and an element upstream of *HML* (primer pair O1/O22; open square). Primer sequences and positions are indicated in Table S1. The amount of PCR product is plotted versus the concentration of DNA. The linear range for PCR amplification is found to the left of the gray line. A template concentration is chosen for all templates within the linear range and is used for all subsequent PCR reactions. (C) Restriction cut sites with primers designed are marked with a hatch mark. Primer names for each restriction site are labeled above the hatch mark. *HML* is depicted as a black box. The centromere is depicted as a black oval and *HMR* is depicted as a gray box.
(1.11 MB TIF)

Figure S2 (A) Analysis of interactions in *MATα* cells examining cross-linking frequencies between the *Eco*RI fragment containing *HML* with other restriction fragments along the length of chromosome III. (B) Analysis of interactions in *MATα* cells examining cross-linking frequencies between the *Eco*RI fragment containing *HMR* with other restriction fragments along the length of chromosome III. (C) Analysis of interactions in *REΔ MATα* mutant cells examining crosslinking frequencies between the *Eco*RI fragment containing *HMR* with other restriction sites along the length of chromosome III. (D) Analysis of interactions in *REΔ MAT* ***a*** cells examining crosslinking frequencies between the *Eco*RI fragment containing *HMR* with other restriction sites along the length of chromosome III. (E) Analysis of interactions in *MAT* ***a*** cells examining cross-linking frequencies between the *Eco*RI fragment containing the left telomere with other restriction fragments along the length of chromosome III. (F) Analysis of interactions in *MAT* ***a*** cells examining cross-linking frequencies between the *Eco*RI fragment containing the right telomere with other restriction fragments along the length of chromosome III. (G) Analysis of interactions in *MATα sir1Δ* cells examining cross-linking frequencies between the *Eco*RI fragment containing the left telomere with other restriction fragments along the length of chromosome III. (H) Analysis of interactions in *MATα sir1Δ* cells examining cross-linking frequencies between the *Eco*RI fragment containing the right telomere with other restriction fragments along the length of chromosome III.
(1.69 MB TIF)

Figure S3 (A) Analysis of interactions in *MATα/MAT* ***a*** cells examining cross-linking frequencies between the *Eco*RI fragment containing *HMR* with other restriction fragments along the length of chromosome III. (B) Analysis of interactions in *MATα rtt109Δ* cells examining cross-linking frequencies between the *Eco*RI fragment containing *HMR* with other restriction fragments along the length of chromosome III. (C) Cutting efficiency of fragments surrounding, as well as, containing *HML*. *Eco*RI digestion of cross-linked chromatin was calculated as the percentage of digested versus undigested chromatin in a fragment upstream of *HML*, the fragment that contains *HML*, and a fragment downstream of *HML*. Error bars represent the standard error of the mean (n = 3). (D) 3C Analysis of interactions between the left telomere of chromosome III (primer O1) with the right telomere of chromosome III (primer O24) and the left telomere of chromosome I (primer O35). Data was normalized to a control interaction which was set at 1 (primer pair O1/O4). In the *yku70Δesc1Δ* mutant the interaction between the left telomere of chromosome III and the left telomere of chromosome 1 is reduced compared to wild type.
(1.12 MB TIF)

Figure S4 Biological repeats show reproducibility of 3C analyses. Analysis of interactions in *MATα*, *sir3Δ*, *esc2Δ*, and *asf1Δ* cells examining crosslinking frequencies between the *Eco*RI fragment containing *HMR* with other restriction fragments along the length of chromosome III.
(0.72 MB TIF)

Table S1 List of primers used in this study, with their location in the genome for *Eco*RI, *Xba*I, and *Aci*I. Any interesting elements located within these fragments are indicated. Primers designed in the opposite direction are noted and marked with an asterisk.
(0.23 MB PDF)

Table S2 Yeast strains used in this study. Strains marked with an asterisk have been previously described in Bystricky et al. (2005; 2009) [30],[47].
(1.51 MB TIF)

Table S3 Values for 3C signal strength, control PCR signal strength, crosslinking frequencies, averaged crosslinking frequencies, standard error of the mean, normalized average crosslinking frequency, and normalized standard error of the mean are listed for *MAT* ***a*** cells. Values for normalized average crosslinking frequency and normalized standard error of the mean are listed for *MATα* cells and all mutants analyzed.
(1.32 MB PDF)

## References

[1] Cremer T, Cremer C (2001). Chromosome territories, nuclear architecture and gene regulation in mammalian cells.. Nat Rev Genet.

[2] Sexton T, Schober H, Fraser P, Gasser SM (2007). Gene regulation through nuclear organization.. Nat Struct Mol Biol.

[3] Fraser P, Bickmore W (2007). Nuclear organization of the genome and the potential for gene regulation.. Nature.

[4] Miele A, Dekker J (2008). Long-range chromosomal interactions and gene regulation.. Mol Bio Syst.

[5] Hilliker AJ, Appels R (1989). The arrangement of interphase chromosomes: structural and functional aspects.. Exp Cell Res.

[6] Haaf T, Schmid M (1991). Chromosome topology in mammalian interphase nuclei.. Exp Cell Res.

[7] Fransz P, De Jong JH, Lysak M, Castiglione MR, Schubert I (2002). Interphase chromosomes in Arabidopsis are organized as well defined chromocenters from which euchromatin loops emanate.. Proc Natl Acad Sci U S A.

[8] Croft JA, Bridger JM, Boyle S, Perry P, Teague P (1999). Differences in the localization and morphology of chromosomes in the human nucleus.. J Cell Biol.

[9] Gilbert N, Gilchrist S, Bickmore WA (2005). Chromatin organization in the mammalian nucleus.. Int Rev Cytol.

[10] Loo S, Rine J (1995). Silencing and heritable domains of gene expression.. Annu Rev Cell Dev Biol.

[11] Rusche LN, Kirchmaier AL, Rine J (2003). The establishment, inheritance, and function of silenced chromatin in Saccharomyces cerevisiae.. Annu Rev Biochem.

[12] Palladino F, Laroche T, Gilson E, Axelrod A, Pillus L (1993). SIR3 and SIR4 proteins are required for the positioning and integrity of yeast telomeres.. Cell.

[13] Gotta M, Laroche T, Formenton A, Maillet L, Scherthan H (1996). The clustering of telomeres and colocalization with Rap1, Sir3, and Sir4 proteins in wild-type Saccharomyces cerevisiae.. J Cell Biol.

[14] Maillet L, Boscheron C, Gotta M, Marcand S, Gilson E (1996). Evidence for silencing compartments within the yeast nucleus: a role for telomere proximity and Sir protein concentration in silencer-mediated repression.. Genes Dev.

[15] Trelles-Sticken E, Dresser ME, Scherthan H (2000). Meiotic telomere protein Ndj1p is required for meiosis-specific telomere distribution, bouquetformation and efficient homologue pairing.. J Cell Biol.

[16] Hediger F, Neumann FR, Van Houwe G, Dubrana K, Gasser SM (2002). Live imaging of telomeres: yKu and Sir proteins define redundant telomere-anchoring pathways in yeast.. Curr Biol.

[17] Funabiki H, Hagan I, Uzawa S, Yanagida M (1993). Cell cycle-dependent specific positioning and clustering of centromeres and telomeres in fission yeast.. J Cell Biol.

[18] Thompson JS, Johnson LM, Grunstein M (1994). Specific repression of the yeast silent mating locus HMR by an adjacent telomere.. Mol Cell Biol.

[19] Andrulis ED, Neiman AM, Zappulla DC, Sternglanz R (1998). Perinuclear localization of chromatin facilitates transcriptional silencing.. Nature.

[20] Haber JE (1998). Mating-type gene switching in Saccharomyces cerevisiae.. Annu Rev Genet.

[21] Fox CA, McConnell KH (2005). Toward biochemical understanding of a transcriptionally silenced chromosomal domain in Saccharomyces cerevisiae.. J Biol Chem.

[22] Tanny JC, Dowd GJ, Huang J, Hilz H, Moazed D (1999). An enzymatic activity in the yeast Sir2 protein that is essential for gene silencing.. Cell.

[23] Imai S, Armstrong CM, Kaeberlein M, Guarente L (2000). Transcriptional silencing and longevity protein Sir2 is an NAD-dependent histone deacetylase.. Nature.

[24] Weiss K, Simpson RT (1998). High-resolution structural analysis of chromatin at specific loci: Saccharomyces cerevisiae silent mating type locus HMLalpha.. Mol Cell Biol.

[25] Ravindra A, Weiss K, Simpson RT (1999). High-resolution structural analysis of chromatin at specific loci: Saccharomyces cerevisiae silent mating-type locus HMRa.. Mol Cell Biol.

[26] Enomoto S, Berman J (1998). Chromatin assembly factor I contributes to the maintenance, but not the re-establishment, of silencing at the yeast silent mating loci.. Genes Dev.

[27] Sharp JA, Fouts ET, Krawitz DC, Kaufman PD (2001). Yeast histone deposition protein Asf1p requires Hir proteins and PCNA for heterochromatic silencing.. Curr Biol.

[28] Valenzuela L, Dhillon N, Dubey RN, Gartenberg MR, Kamakaka RT (2008). Long-range communication between the silencers of HMR.. Mol Cell Biol.

[29] Dekker J, Rippe K, Dekker M, Kleckner N (2002). Capturing Chromosome Conformation.. Science.

[30] Bystricky K, Laroche T, van Houwe G, Blaszczyk M, Gasser SM (2005). Chromosome looping in yeast: telomere pairing and coordinated movement reflect anchoring efficiency and territorial organization.. J Cell Biol.

[31] Houston PL, Broach JR (2006). The dynamics of homologous pairing during mating type interconversion in budding yeast.. PLoS Genet.

[32] Miele A, Gheldof N, Tabuchi TM, Dostie J, Dekker J, Ausubel FM, Brent R, Kingston RE, Moore DD, Seidman JG, Smith JA, Struhl K (2006). Mapping chromatin interactions by Chromosome Conformation Capture (3C).. Current Protocols in Molecular Biology.

[33] Gheldof N, Tabuchi TM, Dekker J (2006). The active FMR1 promoter is associated with a large domain of altered chromatin conformation with embedded local histone modifications.. Proc Natl Acad Sci U S A.

[34] Dekker J (2007). GC- and AT-rich chromatin domains differ in conformation and histone modification status and are differentially modulated by Rpd3p.. Genome Biol.

[35] Dostie J, Richmond TA, Arnaout RA, Selzer RR, Lee WL (2006). Chromosome Conformation Capture Carbon Copy (5C): A Massively Parallel Solution for Mapping Interactions between Genomic Elements.. Genome Res.

[36] Dekker J (2008). Mapping in vivo chromatin interactions in yeast suggests an extended chromatin fiber with regional variation in compaction.. J Biol Chem.

[37] Splinter E, Grosveld F, de Laat W (2004). 3C technology: analyzing the spatial organization of genomic loci in vivo.. Methods Enzymol.

[38] Dekker J (2006). The 3 C's of Chromosome Conformation Capture: Controls, Controls, Controls.. Nat Methods.

[39] Tolhuis B, Palstra RJ, Splinter E, Grosveld F, de Laat W (2002). Looping and Interaction between Hypersensitive Sites in the Active beta-globin Locus.. Mol Cell.

[40] Haber JE (1998). A locus control region regulates yeast recombination.. Trends Genet.

[41] Aparicio OM, Billington BL, Gottschling DE (1991). Modifiers of position effect are shared between telomeric and silent mating-type loci in S. cerevisiae.. Cell.

[42] Pillus L, Rine J (1989). Epigenetic inheritance of transcriptional states in S. cerevisiae.. Cell.

[43] Dhillon N, Kamakaka RT (2000). A histone variant, Htz1p, and a Sir1p-like protein, Esc2p, mediate silencing at HMR.. Mol Cell.

[44] Cuperus G, Shore D (2002). Restoration of silencing in Saccharomyces cerevisiae by tethering of a novel Sir2-interacting protein, Esc8.. Genetics.

[45] Yang B, Kirchmaier AL (2006). Bypassing the catalytic activity of SIR2 for SIR protein spreading in Saccharomyces cerevisiae.. Mol Biol Cell.

[46] Gartenberg MR, Neumann FR, Laroche T, Blaszczyk M, Gasser SM (2004). Sir-mediated repression can occur independently of chromosomal and subnuclear contexts.. Cell.

[47] Bystricky K, Van Attikum H, Montiel MD, Dion V, Gehlen L (2009). Regulation of nuclear positioning and dynamics of the silent mating type loci by the yeast Ku70/80 complex.. Mol Cell Biol.

[48] Taddei A, Hediger F, Neumann FR, Bauer C, Gasser SM (2004). Separation of silencing from perinuclear anchoring functions in yeast Ku80, Sir4 and Esc1 proteins.. EMBO J.

[49] Laroche T, Martin SG, Gotta M, Gorham HC, Pryde FE (1998). Mutation of yeast Ku genes disrupts the subnuclear organization of telomeres.. Curr Biol.

[50] Hediger F, Taddei A, Neumann FR, Gasser SM (2004). Methods for visualizing chromatin dynamics in living yeast.. Methods Enzymol.

[51] Maillet L, Gaden F, Brevet V, Fourel G, Martin SG (2001). Ku-deficient yeast strains exhibit alternative states of silencing competence.. EMBO Rep.

[52] Kaufman PD, Kobayashi R, Stillman B (1997). Ultraviolet radiation sensitivity and reduction of telomeric silencing in Saccharomyces cerevisiae cells lacking chromatin assembly factor I.. Genes Dev.

[53] Moshkin YM, Armstrong JA, Maeda RK, Tamkun JW, Verrijzer P (2002). Histone chaperone ASF1 cooperates with the Brahma chromatin-remodelling machinery.. Genes Dev.

[54] Zhang R, Poustovoitov MV, Ye X, Santos HA, Chen W (2005). Formation of MacroH2A-containing senescence-associated heterochromatin foci and senescence driven by ASF1a and HIRA.. Dev Cell.

[55] Goodfellow H, Krejcí A, Moshkin Y, Verrijzer CP, Karch F (2007). Gene-specific targeting of the histone chaperone asf1 to mediate silencing.. Dev Cell.

[56] Kamakaka RT, Bulger M, Kaufman PD, Stillman B, Kadonaga JT (1996). Postreplicative chromatin assembly by Drosophila and human chromatin assembly factor 1.. Mol Cell Biol.

[57] Kaufman PD, Cohen JL, Osley MA (1998). Hir proteins are required for position-dependent gene silencing in Saccharomyces cerevisiae in the absence of chromatin assembly factor I.. Mol Cell Biol.

[58] Tyler JK, Collins KA, Prasad-Sinha J, Amiott E, Bulger M (2001). Interaction between the Drosophila CAF-1 and ASF1 chromatin assembly factors.. Mol Cell Biol.

[59] Krawitz DC, Kama T, Kaufman PD (2002). Chromatin assembly factor I mutants defective for PCNA binding require Asf1/Hir proteins for silencing.. Mol Cell Biol.

[60] Hiraga S, Botsios S, Donaldson AD (2008). Histone H3 lysine 56 acetylation by Rtt109 is crucial for chromosome positioning.. J Cell Biol.

[61] Recht J, Tsubota T, Tanny JC, Diaz RL, Berger JM (2006). Histone chaperone Asf1 is required for histone H3 lysine 56 acetylation, a modification associated with S phase in mitosis and meiosis.. Proc Natl Acad Sci U S A.

[62] Tsubota T, Berndsen CE, Erkmann JA, Smith CL, Yang L (2007). Histone H3-K56 acetylation is catalyzed by histone chaperone-dependent complexes.. Mol Cell.

[63] Xu F, Zhang Q, Zhang K, Xie W, Grunstein M (2007). Sir2 deacetylates histone H3 lysine 56 to regulate telomeric heterochromatin structure in yeast.. Mol Cell.

[64] Schneider J, Bajwa P, Johnson FC, Bhaumik SR, Shilatifard A (2006). Rtt109 is required for proper H3K56 acetylation: a chromatin mark associated with the elongating RNA polymerase II.. J Biol Chem.

[65] Driscoll R, Hudson A, Jackson SP (2007). Yeast Rtt109 promotes genome stability by acetylating histone H3 on lysine 56.. Science.

[66] Huang S, Zhou H, Katzmann D, Hochstrasser M, Atanasova E (2005). Rtt106p is a histone chaperone involved in heterochromatin-mediated silencing.. Proc Natl Acad Sci U S A.

[67] Tyler JK, Adams CR, Chen SR, Kobayashi R, Kamakaka RT (1999). The RCAF complex mediates chromatin assembly during DNA replication and repair.. Nature.

[68] Donze D, Adams CR, Rine J, Kamakaka RT (1999). The boundaries of the silenced HMR domain in Saccharomyces cerevisiae.. Genes Dev.

[69] Triolo T, Sternglanz R (1996). Role of interactions between the origin recognition complex and SIR1 in transcriptional silencing.. Nature.

[70] Gardner KA, Rine J, Fox CA (1999). A region of the Sir1 protein dedicated to recognition of a silencer and required for interaction with the Orc1 protein in saccharomyces cerevisiae.. Genetics.

[71] Suter B, Tong A, Chang M, Yu L, Brown GW (2004). The origin recognition complex links replication, sister chromatid cohesion and transcriptional silencing in Saccharomyces cerevisiae.. Genetics.

[72] Franco AA, Lam WM, Burgers PM, Kaufman PD (2005). Histone deposition protein Asf1 maintains DNA replisome integrity and interacts with replication factor C.. Genes Dev.

[73] Wu X, Haber JE (1996). A 700 bp cis-acting region controls mating-type dependent recombination along the entire left arm of yeast chromosome III.. Cell.

[74] Bressan DA, Vazquez J, Haber JE (2004). Mating type-dependent constraints on the mobility of the left arm of yeast chromosome III.. J Cell Biol.

[75] Taddei A, Van Houwe G, Nagai S, Erb I, van Nimwegen EJ (2009). The functional importance of telomere clustering: Global changes in gene expression result from SIR factor dispersion.. Genome Res in press.

[76] Wach A, Brachat A, Pohlmann R, Philippsen P (1994). New heterologous modules for classical or PCR-based gene disruptions in Saccharomyces cerevisiae.. Yeast.

[77] Bystricky K, Heun P, Gehlen L, Langowski J, Gasser SM (2004). Long-range compaction and flexibility of interphase chromatin in budding yeast analyzed by high-resolution imaging techniques.. Proc Natl Acad Sci U S A.

[78] Heun P, Laroche T, Raghuraman MK, Gasser SM (2001). The positioning and dynamics of origins of replication in the budding yeast nucleus.. J Cell Biol.

[79] Dubrana K, van Attikum H, Hediger F, Gasser SM (2007). The processing of double-strand breaks and binding of single-strand-binding proteins RPA and Rad51 modulate the formation of ATR-kinase foci in yeast.. J Cell Sci.

[80] Schober H, Kalck V, Vega-Palas MA, Van Houwe G, Sage D (2008). Controlled Exchange of Chromosomal Arms Reveals Principles Driving Telomere Interactions in Yeast.. Genome Res.

[81] Splinter E, Heath H, Kooren J, Palstra RJ, Klous P (2004). CTCF mediates long-range chromatin looping and local histone modification in the beta-globin locus.. Genes Dev.

[82] Hagège H, Klous P, Braem C, Splinter E, Dekker J (2007). Quantitative analysis of Chromosome Conformation Capture assays (3C-qPCR).. Nat Protoc.

[83] Smeal T, Claus J, Kennedy B, Cole F, Guarente L (1996). Loss of transcriptional silencing causes sterility in old mother cells of S. cerevisiae.. Cell.

