# Supplementary figures and images for: Yeast Silent Mating Type Loci Form Heterochromatic Clusters through Silencer Protein-Dependent Long-Range Interactions
Source: PLoS Genet. 2009 May 8;5(5):e1000478. doi: 10.1371/journal.pgen.1000478 (PMC2673037; doi:10.1371/journal.pgen.1000478)

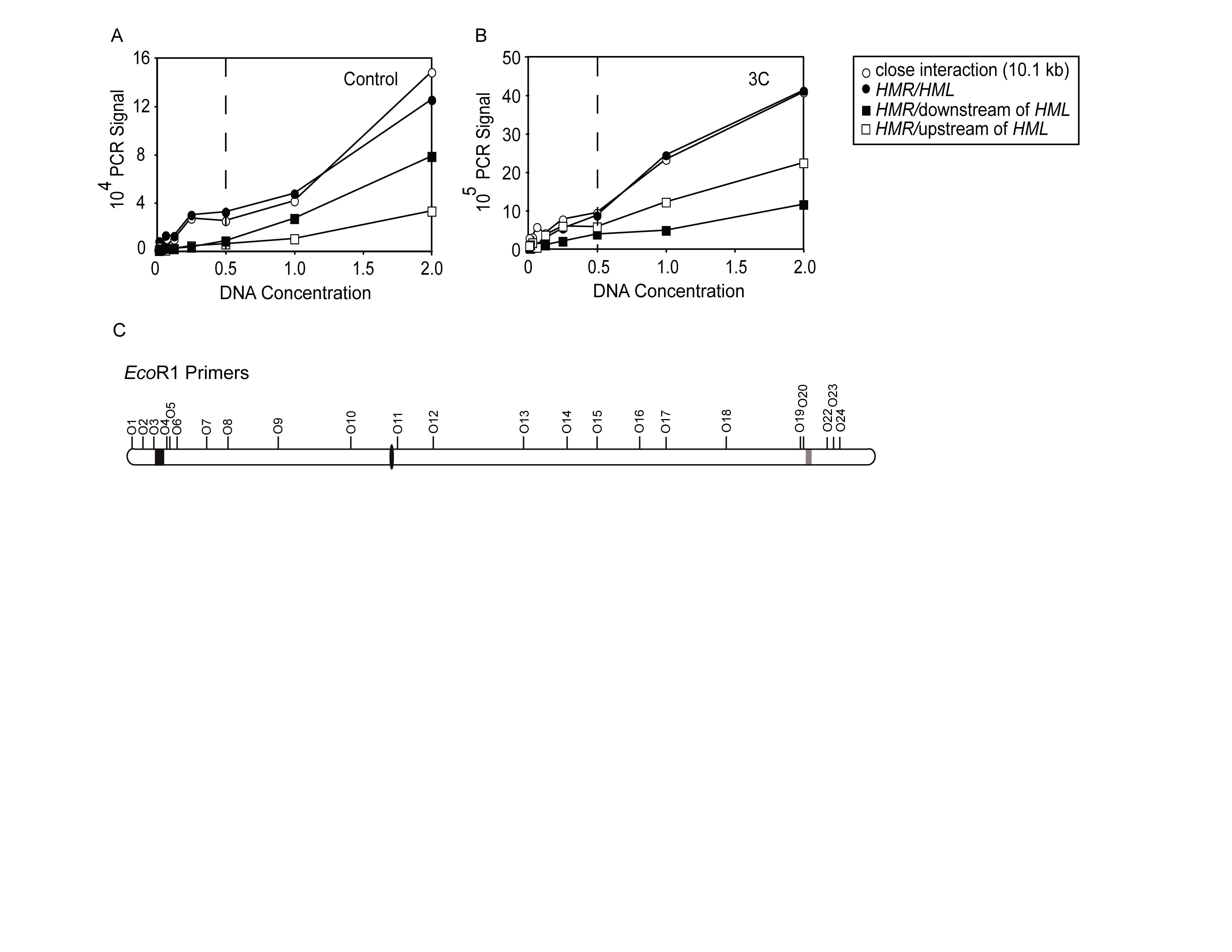

Supplement: Figure S1 — (A) The left (control template) and (B) right (3C template) panels represent titrations using four primer combinations: a close interaction (primer pair O16/O17; 10.1 kb- open circle), interaction between HML and HMR (primer pair O4/O22; filled circle), interaction between HMR and an element downstream of HML (primer pair O7/O22; black square); and an interaction between HMR and an element upstream of HML (primer pair O1/O22; open square). Primer sequences and positions are indicated in Table S1. The amount of PCR product is plotted versus the concentration of DNA. The linear range for PCR amplification is found to the left of the gray line. A template concentration is chosen for all templates within the linear range and is used for all subsequent PCR reactions. (C) Restriction cut sites with primers designed are marked with a hatch mark. Primer names for each restriction site are labeled above the hatch mark. HML is depicted as a black box. The centromere is depicted as a black oval and HMR is depicted as a gray box. (1.11 MB TIF) [file pgen.1000478.s001.tif]

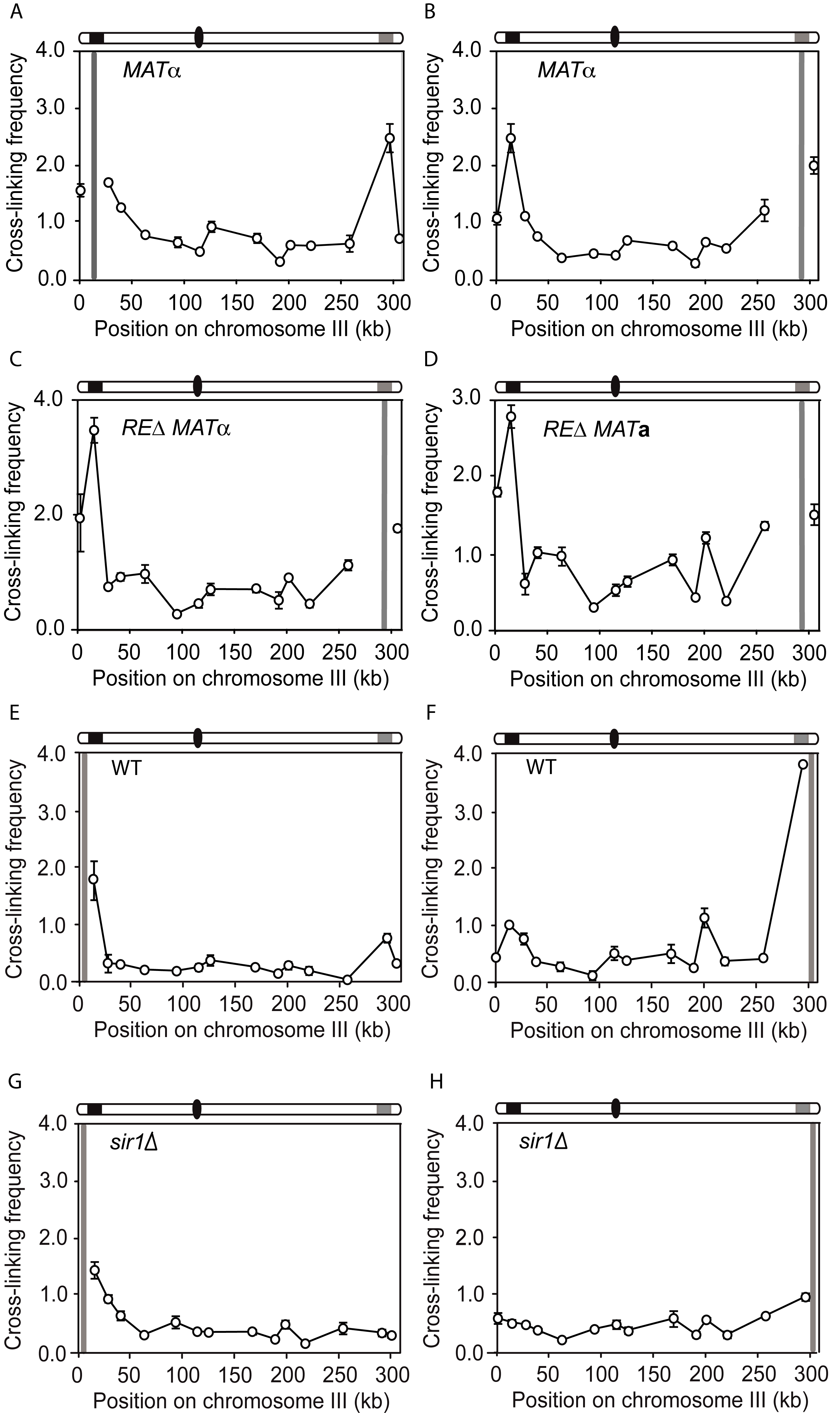

Supplement: Figure S2 — (A) Analysis of interactions in MATα cells examining cross-linking frequencies between the EcoRI fragment containing HML with other restriction fragments along the length of chromosome III. (B) Analysis of interactions in MATα cells examining cross-linking frequencies between the EcoRI fragment containing HMR with other restriction fragments along the length of chromosome III. (C) Analysis of interactions in REΔ MATα mutant cells examining crosslinking frequencies between the EcoRI fragment containing HMR with other restriction sites along the length of chromosome III. (D) Analysis of interactions in REΔ MAT a cells examining crosslinking frequencies between the EcoRI fragment containing HMR with other restriction sites along the length of chromosome III. (E) Analysis of interactions in MAT a cells examining cross-linking frequencies between the EcoRI fragment containing the left telomere with other restriction fragments along the length of chromosome III. (F) Analysis of interactions in MAT a cells examining cross-linking frequencies between the EcoRI fragment containing the right telomere with other restriction fragments along the length of chromosome III. (G) Analysis of interactions in MATα sir1Δ cells examining cross-linking frequencies between the EcoRI fragment containing the left telomere with other restriction fragments along the length of chromosome III. (H) Analysis of interactions in MATα sir1Δ cells examining cross-linking frequencies between the EcoRI fragment containing the right telomere with other restriction fragments along the length of chromosome III. (1.69 MB TIF) [file pgen.1000478.s002.tif]

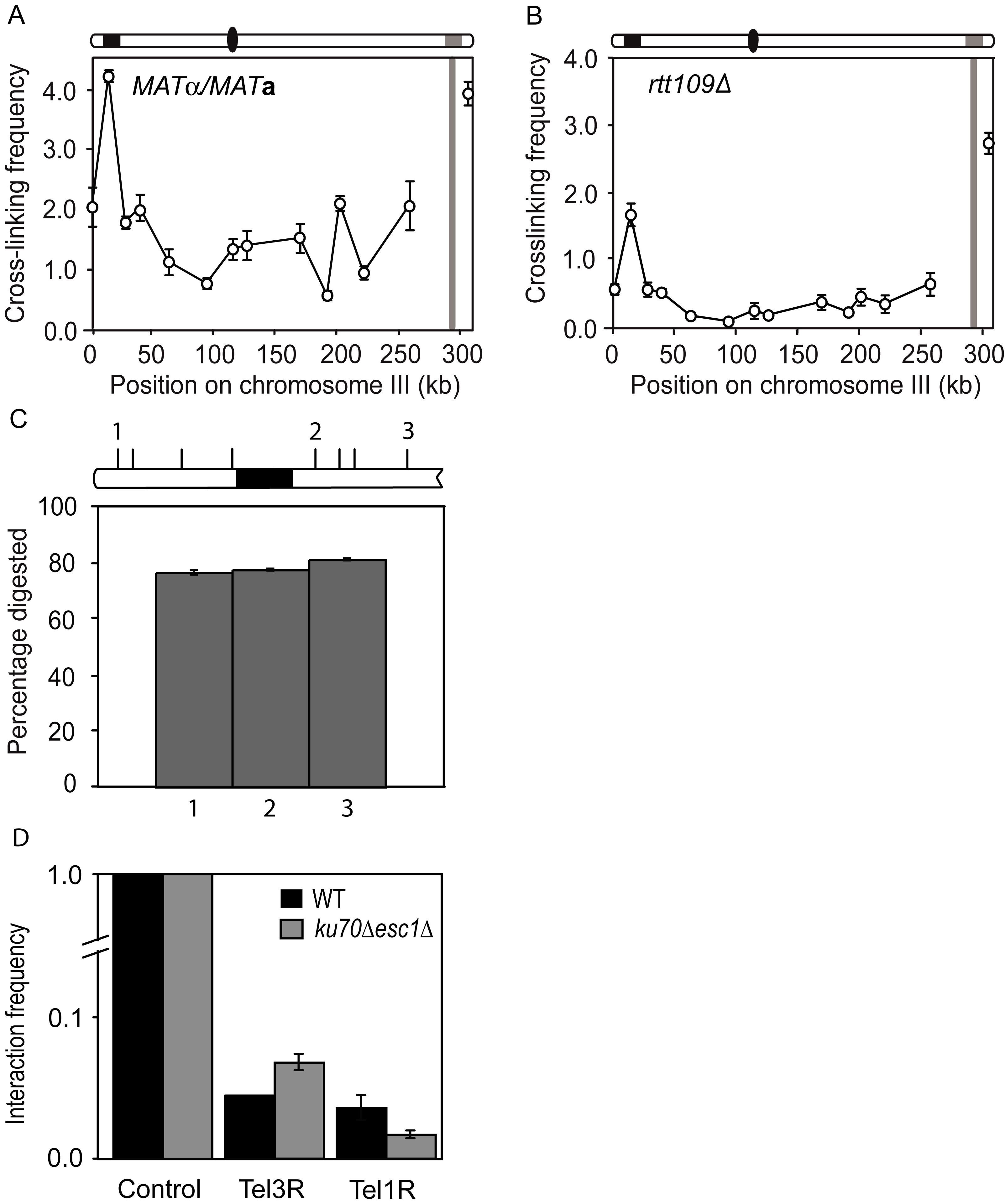

Supplement: Figure S3 — (A) Analysis of interactions in MATα/MAT a cells examining cross-linking frequencies between the EcoRI fragment containing HMR with other restriction fragments along the length of chromosome III. (B) Analysis of interactions in MATα rtt109Δ cells examining cross-linking frequencies between the EcoRI fragment containing HMR with other restriction fragments along the length of chromosome III. (C) Cutting efficiency of fragments surrounding, as well as, containing HML. EcoRI digestion of cross-linked chromatin was calculated as the percentage of digested versus undigested chromatin in a fragment upstream of HML, the fragment that contains HML, and a fragment downstream of HML. Error bars represent the standard error of the mean (n = 3). (D) 3C Analysis of interactions between the left telomere of chromosome III (primer O1) with the right telomere of chromosome III (primer O24) and the left telomere of chromosome I (primer O35). Data was normalized to a control interaction which was set at 1 (primer pair O1/O4). In the yku70Δesc1Δ mutant the interaction between the left telomere of chromosome III and the left telomere of chromosome 1 is reduced compared to wild type. (1.12 MB TIF) [file pgen.1000478.s003.tif]

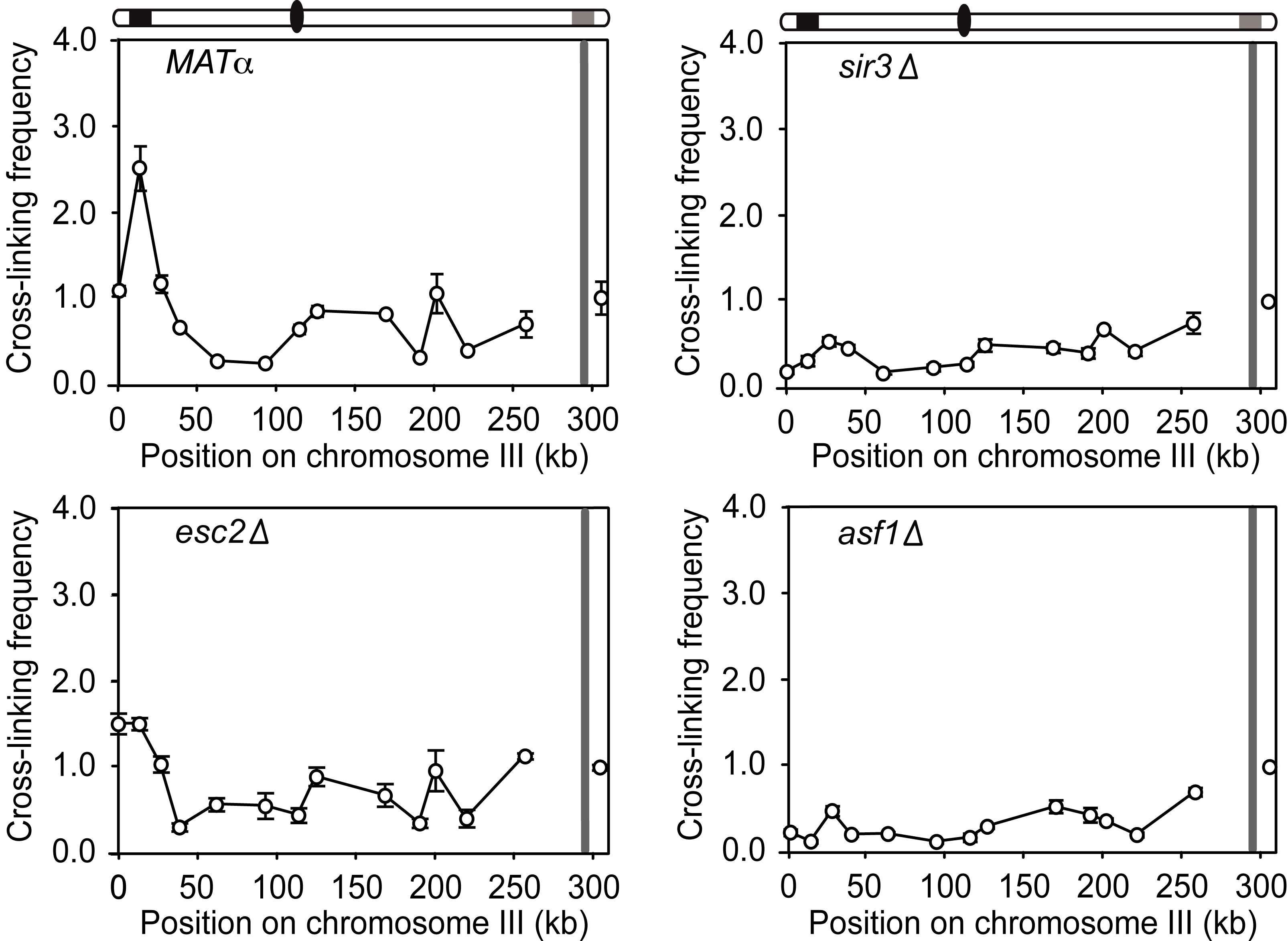

Supplement: Figure S4 — Biological repeats show reproducibility of 3C analyses. Analysis of interactions in MATα, sir3Δ, esc2Δ, and asf1Δ cells examining crosslinking frequencies between the EcoRI fragment containing HMR with other restriction fragments along the length of chromosome III. (0.72 MB TIF) [file pgen.1000478.s004.tif]

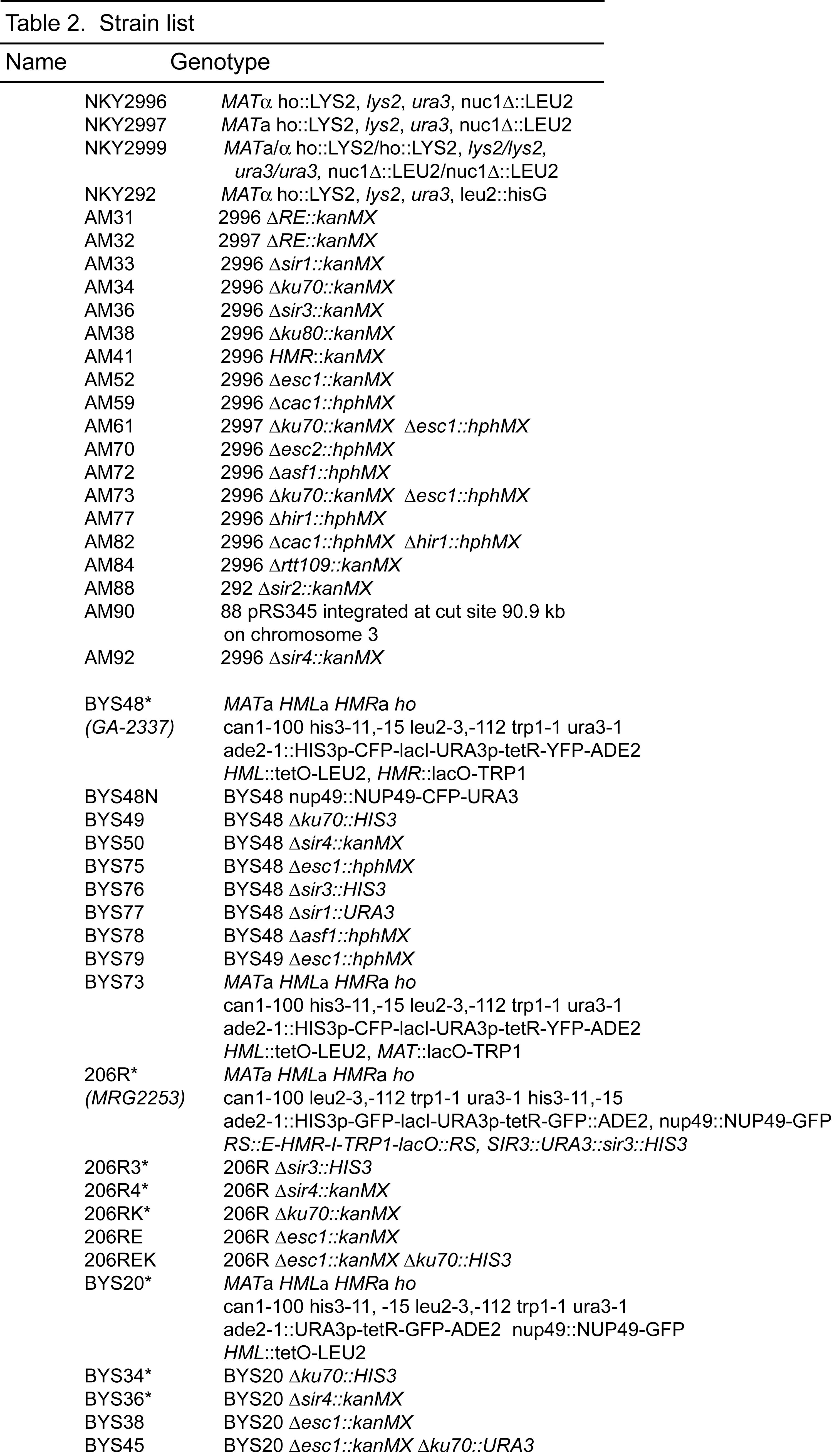

Supplement: Table S2 — Yeast strains used in this study. Strains marked with an asterisk have been previously described in Bystricky et al. (2005; 2009) [30],[47]. (1.51 MB TIF) [file pgen.1000478.s006.tif]
