# Supplementary material for: Yeast Silent Mating Type Loci Form Heterochromatic Clusters through Silencer Protein-Dependent Long-Range Interactions
Source: PLoS Genet. 2009 May 8;5(5):e1000478. doi: 10.1371/journal.pgen.1000478 (PMC2673037; doi:10.1371/journal.pgen.1000478)
Supplement: Table S1 — List of primers used in this study, with their location in the genome for EcoRI, XbaI, and AciI. Any interesting elements located within these fragments are indicated. Primers designed in the opposite direction are noted and marked with an asterisk. (0.23 MB PDF) [file pgen.1000478.s005.pdf]

| *Eco*RI primers | | | | |
|---|---|---|---|---|
| Primer name | Alternative name | Sequence | Coordinates | Feature |
| O1 | JD74 | GGTTATGAGCCCTAGAGCAATGCTTCTG | Chr3:1430-1458 | Left telomere |
| O2 | AM53 | ACCAGCTTGGGGATCAACATCCTAATTT | Chr3:6466-6493 | |
| O3 | AM34 | GGATCAACACAGTATCTTATGAATGGGT | Chr3:11220-11247 | |
| O4 | JD75 | CATGGTAGAGACAGTCTTTGACATGCTG | Chr3:16627-16654 | *HML* |
| O5 | AM54 | GTTAGATCGGAATCTGTTCGAGACAAGT | Chr3:17479-17506 | |
| O6 | AM55 | GCGTCTGCGTGAATGAAGAAGAGACATT | Chr3:21731-21758 | |
| O7 | JD96 | AATGCACCAGAAACAAGATGAAGCAGATTC | Chr3:33248-33277 | Recombination enhancer |
| O8 | JD98 | GTCAAGAGTCTGAACACAAACTCACCGA | Chr3:42342-42369 | |
| O9 | JD77 | GATCTCCTGTTTTTTACGTTCTCTGACCTG | Chr3:64759-64788 | |
| O10 | JD78 | GGTGAGTTCTCAAGTAGTTCTTCCAGGG | Chr3:95300-95327 | |
| O11 | JD79 | CGCCAATCCAGCAATACCTGTACCTCTA | Chr3:117225-117252 | Centromere |
| O12 | JD80 | CTCGTTATTAGTAGGTCGTGCTCTTAAAAG | Chr3:132248-132277 | |
| O13 | JD81 | TGGTTTGGCCATCTCTTCTTGGTTGCGA | Chr3:173262-173289 | |
| O14 | JD82 | GGTGGCACTTCTTCCTCTTGTTCTTATAG | Chr3:192214-192242 | |
| O15 | JD123 | CTTCCTCTTCATCGTGCTCAGGCGTATC | Chr3:204568-204595 | Mating type locus |
| O16 | JD83 | AGCCATTCGCCGGTTTGGTTGACGCTAA | Chr3:222275-222302 | |
| O17 | JD125 | GCCGAAGATCAATTTTACGTCTATGACTCA | Chr3:232314-232353 | |
| O18 | JD182 | CACCGCTACCAGTAGCAAAAACGTTATATTCTG | Chr3:260355-260387 | |
| O19 | AM29 | CTGGTCCTCACAGTTCGCAGGATAACTA | Chr3:290553-290580 | |
| O20 | AM36 | AATGGCTAT ATACATCAAGTCTGGAGGC | Chr3:291233-291260 | |
| O21 | AM144 | CATCACCAATCCTTGCATTTCGTTTCCA | Chr3:291482-291509* | |
| O22 | AM31 | CCAGAAGAATTATGGGCCACCCTCCATG | Chr3:301581-301608 | *HMR* |
| O23 | AM37 | GTTATATCGTAGTTTCCATTCAGCCTGC | Chr3:303919-303946 | |
| O24 | AM32 | GGCGGTTTCTCGAAACATTTCACGATTT | Chr3:306606-306633 | Right telomere |
| O25 | JD277 | AGCCCACTATTTATTGGCTAACAGTGGC | Chr6:150214-150241 | |
| O26 | JD279 | GTTGCTGTCGAATATTTGGTTTTAATAACTC | Chr6:151664-151694 | |
| O27 | JD280 | CCGCGTTGCCCATCGAACTGAACGAGTTAC | Chr6:156905-156934 | |
| O28 | JD281 | CAAAAGATCGCAAAGAAAGTCGCCAAGAGA | Chr6:162920-162949 | |
| O29 | JD282 | TTCGATGTCTTCTTTTGAAGTTGCAGCAAC | Chr6:177991-178020 | |
| O30 | JD283 | AATAACAAGTCATCCTCTATTCGATTGAGC | Chr6:183420-183449 | |
| O31 | JD290 | ACGATTTCTTGAAGAACGAGCCCTTGACT | Chr6:202320-202348 | |
| O32 | JD286 | CCCCTTTTAATTATCGTTTCAGCAGCACCA | Chr6:209230-209259 | |
| O33 | JD288 | GACGCCAACATCCCATTACATGAAAATCAG | Chr6:235825-235854 | |
| O34 | JD292 | AGCGAGGTTTAACCTTTCATACAATTTACGG | Chr6:237970-238000 | |
| O35 | JD153 | GATCCTTACAGGTTTTAAGGTTGCAAAGGG | Chr1:6761-6790 | Left telomere |
| *Xba*I primers | | | | |
| O36 | Xba2 | CCTAAGGCATAAAACCCTAGATCCATCCA | Chr3:10966-10994* | *HML-E* |
| O37 | Xba3 | AGTTGCAATTTCTCTTTGTCAATCAGCTGA | Chr3:20020-20049 | *HML-I* |
| O38 | Xba10 | GCGCAGGTACTCCTGGTTTTTGTTAAAAC | Chr3:292906-292934 | *HMR-E* |
| O39 | Xba29 | CCTTACAGAGGACACCGGTTTACAAAAG | Chr3:293394-293421* | *HMR-I* |

| *Aci*I primers | | | | |
|---|---|---|---|---|
| Primer name | Alternative name | Sequence | Coordinates | Feature |
| O40 | Ac21 | CTCCCCTAGATTTATGTTTCCTATTGCTCT | Chr3:4722-4751 | |
| O41 | Ac1 | GTACCGGTTACAATGGGCAAATGATATAC | Chr3:10373-10401 | |
| O42 | Ac3 | CGTCATTCAAACTTGTATTAGACGAGGG | Chr3:11497-11524 | *HML-E* |
| O43 | Ac4 | CCCATTACGAGCTGTAGTAGTGCTGTGA | Chr3:11759-11786 | |
| O44 | Ac5 | GTGACTTATGAATTGTTGTAGAAGGACGTC | Chr3:12124-12153 | |
| O45 | Ac12 | CACAGGATAGCGTCTGGAAGTCAAAATAC | Chr3:13480-13508 | |
| O46 | Ac31 | GCTGGAAATGGCAAACGAAAATACTATGAC | Chr3:14722-14751 | *HML-I* |
| O47 | Ac10 | GAAATTGTCAGCGACTTCTATTACAGGAG | Chr3:15840-15868 | |
| O48 | Ac16 | GTTGCATGTTAACGTCTCTGATATCAGAGC | Chr3:290129-290158 | |
| O49 | Ac17 | CCGTCACTTTTACCATTGGTTCTAGAATGG | Chr3:290398-290427* | *HMR-E* |
| O50 | Ac29 | GTTCTTTCGGGGAAACTGTATAAAACTTCC | Chr3:293744-293773 | |
| O51 | Ac33 | CAAAGAAATGTGGCATTACTCCACTTCAAG | Chr3:294196-294225 | |
| O52 | Ac35 | GTCACTACATTATATAAACAATAGGAATTG | Chr3:294630-294659* | *HMR-I* |
| O53 | Ac20 | CCTGGTAGTCTTGCAAAGGTGTGAGTAC | Chr3:296331-296358 | |

* Primer is designed on reverse strand
