# Supplementary material for: Yeast Silent Mating Type Loci Form Heterochromatic Clusters through Silencer Protein-Dependent Long-Range Interactions
Source: PLoS Genet. 2009 May 8;5(5):e1000478. doi: 10.1371/journal.pgen.1000478 (PMC2673037; doi:10.1371/journal.pgen.1000478)
Supplement: Table S3 — Values for 3C signal strength, control PCR signal strength, crosslinking frequencies, averaged crosslinking frequencies, standard error of the mean, normalized average crosslinking frequency, and normalized standard error of the mean are listed for MAT a cells. Values for normalized average crosslinking frequency and normalized standard error of the mean are listed for MATα cells and all mutants analyzed. (1.32 MB PDF) [file pgen.1000478.s007.pdf]

Primer Pair	Control PCR signal strength						3C PCR signal strength					
*Mat***a** *HMR* Fixed Primer Experiment												
O21/ O1	60538.8	48969.3	64405.1				16113.8	23886	16452			
O21/ O4	77705.4	66216.9	81112.2	73761.2	58110.1	75421.5	91501.1	88447.2	103161	126560	110235	95025.1
O21/ O7	100982	127666	105778				51654.5	48933.1	54524.3			
O21/ O8	168929	148459	165795				50596.8	53270	60648.8			
O21/ O9	151214	140977	168619				10830.7	27663.7	18619.4			
O21/ O10	100433	110389	107721				8544.42	5910.33	19383.8			
O21/ O11	160065	138569	156375				15860.8	29393.8	27757.5			
O21/ O12	77024.3	47201.3	61578.3				18513.9	34382.1	9437.17			
O21/ O13	98488.5	91397.9	127059				44192.1	48585.8	38732.3			
O21/ O14	94391	71637.6	93518.4				21782.4	18703.3	16886.9			
O21/ O15	114195	93512.1	84221.9				44291.3	43195.4	56525.7			
O21/ O16	11724.8	10493.5	9575.23				4336.53	1747.57	3404.64			
O21/ O17	48543.1	50456.5	56121.5				25926.2	29479.6	16050.5			
O21/ O23	249905	238637	274907				336430	327855	339454			
*Mat***a** *HML* Fixed Primer Experiment												
O4/ O1	154491	129867	114495				158103	102749	143418			
O4/ O7	185357	151119	223988				314141	312650	325642			
O4/ O8	229973	206611	223258				129100	157262	139400			
O4/ O9	196974	186594	204622				33605.8	37587.6	49549.1			
O4/ O10	148815	108738	113740				51913.8	41149.6	33507.9			
O4/ O11	212870	179893	195216				62251.5	46236.3	49611			
O4/ O12	62912.9	66065.3	51008.5				21099.3	31945.3	24242.8			
O4/ O13	149706	107306	154094				56193.6	34326.6	88593.8			
O4/ O14	87339.8	93499.4	85937.1				25646.5	15732.5	16065.8			
O4/ O15	144616	137201	162591				51683.1	79217.8	53327.4			
O4/ O16	152253	166502	142869				53510.6	28824.7	32438.2			
O4/ O17	89632.6	103940	106365				21213.3	18838	23274.3			
O4/ O21	77705.4	66216.9	81112.2	73761.2	58110.1	75421.5	91501.1	88447.2	103161	126560	110235	95025.1
O4/ O23	302165	311542	277550				131714	125697	149231			

Primer Pair	Interaction Frequencies						Averaged Interaction Frequencies	Standard Error of the Mean
*Mat***a** *HMR* Fixed Primer Experiment								
O21/ O1	0.266173099	0.487775	0.255446				0.336464567	0.075718566
O21/ O4	1.177538498	1.335719	1.271831	1.715807	1.897002	1.259921	1.442969837	0.119071105
O21/ O7	0.511521855	0.38329	0.51546				0.470090528	0.043415153
O21/ O8	0.299515181	0.35882	0.365806				0.341380251	0.021029466
O21/ O9	0.071624982	0.196228	0.110423				0.126092117	0.036813268
O21/ O10	0.085075822	0.053541	0.179944				0.10618708	0.037985649
O21/ O11	0.099089745	0.212124	0.177506				0.162906555	0.033436702
O21/ O12	0.240364404	0.728414	0.153255				0.374011154	0.178976899
O21/ O13	0.448703148	0.531586	0.304837				0.428375263	0.066241039
O21/ O14	0.230767764	0.261082	0.180573				0.224140985	0.02347599
O21/ O15	0.387856736	0.461923	0.671152				0.506977295	0.084826228
O21/ O16	0.369859614	0.166538	0.355567				0.297321795	0.065521757
O21/ O17	0.534086204	0.584258	0.285996				0.468113165	0.092203418
O21/ O23	1.346231568	1.373865	1.234796				1.318297414	0.042506055
*Mat***a** *HML* Fixed Primer Experiment								
O4/ O1	1.023380003	0.791186	1.252614				1.022393341	0.133203497
O4/ O7	1.694788975	2.068899	1.453837				1.739175044	0.178934878
O4/ O8	0.561370248	0.76115	0.62439				0.648970049	0.058966516
O4/ O9	0.170610334	0.201441	0.242149				0.204733441	0.020717084
O4/ O10	0.348847898	0.378429	0.294601				0.340625876	0.024545783
O4/ O11	0.292439047	0.257021	0.254134				0.267864683	0.012315418
O4/ O12	0.335373191	0.483541	0.47527				0.431394761	0.048070125
O4/ O13	0.375359705	0.319895	0.574933				0.423395898	0.077442078
O4/ O14	0.293640471	0.168263	0.186948				0.216283981	0.039052548
O4/ O15	0.357381618	0.577385	0.327985				0.420917189	0.078692808
O4/ O16	0.351458428	0.173119	0.227049				0.250542075	0.052805225
O4/ O17	0.236669471	0.181239	0.218815				0.212241349	0.016335473
O4/ O21	1.177538498	1.335719	1.271831	1.715807	1.897002	1.259921	1.442969837	0.119071105
O4/ O23	0.435900915	0.403467	0.537672				0.459013559	0.040428563

Primer Pair	Normalized Interaction Frequencies	Normalized Standard Error of the Mean
***MAT*a *HMR* Fixed Primer Experiment**		
O21/ O1	0.754646863	0.16982703
O21/ O4	3.23639624	0.267061215
O21/ O7	1.05435275	0.097374619
O21/ O8	0.765672111	0.047166395
O21/ O9	0.282808443	0.082567437
O21/ O10	0.238163999	0.085196937
O21/ O11	0.365378504	0.074994234
O21/ O12	0.838859041	0.401422226
O21/ O13	0.960790763	0.148570154
O21/ O14	0.502719476	0.052653635
O21/ O15	1.137085038	0.190254349
O21/ O16	0.666854646	0.146956896
O21/ O17	1.049917779	0.206800439
O21/ O23	2.956771989	0.095335629
***MAT*a *HML* Fixed Primer Experiment**		
O4/ O1	2.29309711	0.298758356
O4/ O7	3.900746522	0.401327978
O4/ O8	1.455556571	0.13225433
O4/ O9	0.459190844	0.046465762
O4/ O10	0.763980143	0.055053041
O4/ O11	0.600786121	0.027621903
O4/ O12	0.967563109	0.107815124
O4/ O13	0.949622686	0.173692648
O4/ O14	0.485097225	0.087589855
O4/ O15	0.944063259	0.176497874
O4/ O16	0.561933734	0.118435346
O4/ O17	0.476030119	0.036638369
O4/ O21	3.23639624	0.267061215
O4/ O23	1.02950853	0.090676081
MATα *HMR* Fixed Primer Experiment		
O21/ O1	1.214865585	0.118091808
O21/ O4	2.794702901	0.27958518
O21/ O7	1.261206851	0.041851338
O21/ O8	0.864650394	0.022407296
O21/ O9	0.442962144	0.034527429
O21/ O10	0.52595912	0.063359638
O21/ O11	0.494844624	0.05922577
O21/ O12	0.78421554	0.053137293
O21/ O13	0.680436588	0.057537219
O21/ O14	0.335020938	0.071196142
O21/ O15	0.749305998	0.061413099
O21/ O16	0.628236457	0.057139945
O21/ O17	1.374750579	0.21320943
O21/ O23	2.258131419	0.16367818

Primer Pair	Normalized Interaction Frequencies	Normalized Standard Error of the Mean
*sir1*Δ *HMR* Fixed Primer Experiment		
O21/ O1	0.889453698	0.106405563
O21/ O4	0.916042824	0.115790844
O21/ O7	0.936014244	0.123084741
O21/ O8	0.797406672	0.150969514
O21/ O9	0.167759772	0.006325723
O21/ O10	0.34975848	0.046465448
O21/ O11	0.353675266	0.094927237
O21/ O12	0.709922904	0.231043042
O21/ O13	1.080012749	0.484424248
O21/ O14	0.375721748	0.068212953
O21/ O15	1.118542579	0.216745304
O21/ O16	0.421192566	0.061082786
O21/ O17	1.43057509	0.470669287
O21/ O23	2.612939347	0.237885866
*sir3*Δ *HMR* Fixed Primer Experiment		
O21/ O1	0.747902445	0.279868999
O21/ O4	0.556830157	0.059767508
O21/ O7	0.603750019	0.1014268
O21/ O8	0.606765557	0.085691664
O21/ O9	0.182052722	0.044664444
O21/ O10	0.273942967	0.055004915
O21/ O11	0.782560857	0.042223271
O21/ O12	0.661561695	0.293566574
O21/ O13	0.883912079	0.103359112
O21/ O14	0.631471603	0.161541057
O21/ O15	1.316493292	0.285320514
O21/ O16	0.36274008	0.039530688
O21/ O17	1.080879267	0.025414029
O21/ O23	2.536595832	0.043826452
*MAT***a***/MAT*α *HMR* Fixed Primer Experiment		
O21/ O1	2.030007338	0.324894478
O21/ O4	4.211005437	0.097896565
O21/ O7	1.774069126	0.099921691
O21/ O8	2.019810671	0.217262545
O21/ O9	1.114447132	0.215485015
O21/ O10	0.757278634	0.087753502
O21/ O11	1.328236971	0.172604967
O21/ O12	1.390234658	0.24465591
O21/ O13	1.509123088	0.238064933
O21/ O14	0.55575908	0.076325003
O21/ O15	2.091197933	0.123976444
O21/ O16	0.933715209	0.108707962
O21/ O17	2.054015213	0.406111061
O21/ O23	3.920603613	0.195390879

Primer Pair	Normalized Interaction Frequencies	Normalized Standard Error of the Mean
*esc2*Δ *HMR* Fixed Primer Experiment		
O21/ O1	1.004785279	0.112802508
O21/ O4	0.712966753	0.060551227
O21/ O7	0.221183188	0.086580933
O21/ O8	0.279774653	0.031348684
O21/ O9	0.277577574	0.026253229
O21/ O10	0.178141676	0.047658764
O21/ O11	0.297988984	0.023857616
O21/ O12	0.349601979	0.106795329
O21/ O13	0.519832475	0.232725427
O21/ O14	0.300996965	0.032336955
O21/ O15	0.529375156	0.136628662
O21/ O16	0.299273771	0.096054453
O21/ O17	1.110035257	0.296451031
O21/ O23	2.710878412	0.258580483
*cac1*Δ *HMR* Fixed Primer Experiment		
O21/ O1	1.041546095	0.307455062
O21/ O4	2.245902552	0.353179599
O21/ O7	0.77669006	0.132603861
O21/ O8	0.614529213	0.144676499
O21/ O9	0.260087634	0.090156381
O21/ O10	0.47453408	0.234166659
O21/ O11	0.326526697	0.082100185
O21/ O12	0.476650441	0.039653599
O21/ O13	0.687388649	0.124858064
O21/ O14	0.262903277	0.078819527
O21/ O15	0.61700196	0.030902971
O21/ O16	0.18863832	0.117437825
O21/ O17	0.965492647	0.3511845
O21/ O23	1.870012281	0.08321961
*hir1*Δ *HMR* Fixed Primer Experiment		
O21/ O1	1.360855006	0.177814651
O21/ O4	3.10225865	0.438851502
O21/ O7	1.070551929	0.089010738
O21/ O8	0.578844707	0.024187957
O21/ O9	0.225441136	0.037183893
O21/ O10	0.290210412	0.042057806
O21/ O11	0.276657437	0.031483641
O21/ O12	0.574128123	0.156021184
O21/ O13	0.579289161	0.075855458
O21/ O14	0.331439488	0.058083272
O21/ O15	0.789087397	0.013723788
O21/ O16	0.535961844	0.058785527
O21/ O17	1.774643114	0.082533428
O21/ O23	1.026023733	0.103508824

Primer Pair	Normalized Interaction Frequencies	Normalized Standard Error of the Mean
cac1Δ hir1Δ HMR Fixed Primer Experiment		
O21/ O1	1.224826196	0.67778452
O21/ O4	1.835980472	0.105500361
O21/ O7	1.217048861	0.391169228
O21/ O8	2.068644332	0.175422599
O21/ O9	1.006008249	0.286830456
O21/ O10	0.948298834	0.153548411
O21/ O11	0.859065157	0.121126586
O21/ O12	1.247385247	0.319589792
O21/ O13	1.882143219	0.265963661
O21/ O14	1.052073105	0.434404305
O21/ O15	2.667957676	0.527180109
O21/ O16	1.403038275	0.27889078
O21/ O17	1.956012518	0.065403888
O21/ O23	4.117038799	0.890005888
asf1 Δ HMR Fixed Primer Experiment		
O21/ O1	0.570217871	0.067379954
O21/ O4	0.374754518	0.047253448
O21/ O7	0.490615719	0.028585487
O21/ O8	0.246883493	0.037146215
O21/ O9	0.377838325	0.064883601
O21/ O10	0.361697131	0.0209399
O21/ O11	0.416589015	0.084319934
O21/ O12	0.194929598	0.027030557
O21/ O13	0.400810063	0.117229549
O21/ O14	0.302959371	0.054280607
O21/ O15	0.66661218	0.028436262
O21/ O16	0.373404133	0.066819371
O21/ O17	1.403388022	0.26294777
O21/ O23	2.194525676	0.196827212
rtt109Δ HMR Fixed Primer Experiment		
O21/ O1	0.567777361	0.077434373
O21/ O4	1.669404345	0.169456826
O21/ O7	0.565504446	0.105521989
O21/ O8	0.515977771	0.056303165
O21/ O9	0.174817357	0.052619841
O21/ O10	0.093802378	0.053974317
O21/ O11	0.250648076	0.119421122
O21/ O12	0.185829679	0.03148394
O21/ O13	0.377112314	0.109109356
O21/ O14	0.226164892	0.049287825
O21/ O15	0.454576136	0.122852676
O21/ O16	0.351779423	0.129884023
O21/ O17	0.645995855	0.167574797
O21/ O23	2.724977684	0.15504539

Primer Pair	Normalized Interaction Frequencies	Normalized Standard Error of the Mean
*ku70*Δ *HMR* Fixed Primer Experiment		
O21/ O1	3.11488268	0.591630546
O21/ O4	8.224625036	0.658538572
O21/ O7	2.831998861	0.135122844
O21/ O8	1.37145975	0.088477871
O21/ O9	0.615306005	0.059039549
O21/ O10	0.929938355	0.048770133
O21/ O11	0.604618848	0.085492668
O21/ O12	1.410430545	0.163735629
O21/ O13	1.603177928	0.123427926
O21/ O14	0.963760846	0.178208293
O21/ O15	1.982471482	0.252699556
O21/ O16	0.88020045	0.070130555
O21/ O17	3.859432137	0.101649791
O21/ O23	2.445604082	0.202140399
*ku80*Δ *HMR* Fixed Primer Experiment		
O21/ O1	2.585944046	0.543276386
O21/ O4	4.033924716	0.331897316
O21/ O7	2.72459153	0.55881201
O21/ O8	1.541285834	0.027917852
O21/ O9	0.735911562	0.08873278
O21/ O10	0.839638463	0.080604433
O21/ O11	1.248509598	0.1033956
O21/ O12	2.083964224	0.315220926
O21/ O13	1.404024163	0.055075306
O21/ O14	1.043448925	0.105070418
O21/ O15	1.923289411	0.095926552
O21/ O16	0.937865642	0.056026547
O21/ O17	3.508873573	0.705799283
O21/ O23	1.732482506	0.088807595
*esc1*Δ *HMR* Fixed Primer Experiment		
O21/ O1	4.012656036	0.704861108
O21/ O4	12.69363694	2.349507423
O21/ O7	4.431103615	0.132626633
O21/ O8	2.941142265	0.417832383
O21/ O9	0.390019367	0.099437854
O21/ O10	0.837790454	0.190193236
O21/ O11	0.691562718	0.159230772
O21/ O12	2.053873867	0.211749142
O21/ O13	1.807306279	0.597107887
O21/ O14	0.571493026	0.10832359
O21/ O15	2.625049813	0.313090104
O21/ O16	0.477943756	0.076527722
O21/ O17	3.946185443	2.081821732

Primer Pair	Normalized Interaction Frequencies	Normalized Standard Error of the Mean
*ku70*Δ*esc1*Δ *HMR* Fixed Primer Experiment		
O21/ O1	7.420006086	0.459701922
O21/ O4	14.72104138	2.264054339
O21/ O7	2.33852603	0.130758456
O21/ O8	2.425791484	0.271752941
O21/ O9	0.076888775	0.007377875
O21/ O10	0.55540987	0.145373356
O21/ O11	0.335912363	0.015666031
O21/ O12	0.818157895	0.17208751
O21/ O13	1.177598434	0.402178499
O21/ O14	0.470668852	0.080577016
O21/ O15	1.485911756	0.21589271
O21/ O16	0.189128288	0.019054167
O21/ O17	3.182863531	1.257499632
O21/ O23	2.53010516	0.28215603
*sir4*Δ *HMR* Fixed Primer Experiment		
O21/ O1	0.075106649	0.003688055
O21/ O4	0.267603313	0.05653249
O21/ O7	0.478640947	0.025872737
O21/ O8	0.537425537	0.081706908
O21/ O9	0.437665684	0.018090483
O21/ O10	0.241786091	0.021113524
O21/ O11	0.631658653	0.022230052
O21/ O12	0.373149312	0.032333722
O21/ O13	0.779906183	0.009932234
O21/ O14	0.419672104	0.026842006
O21/ O15	0.757118807	0.041494685
O21/ O16	0.461382359	0.025681157
O21/ O17	0.910705793	0.016810398
O21/ O23	1.397279357	0.086660012
*sir2*Δ *HMR* Fixed Primer Experiment		
O21/ O1	0.352452926	0.042002346
O21/ O4	0.639999546	0.131781853
O21/ O7	0.97349984	0.042625044
O21/ O8	0.733135223	0.022230052
O21/ O9	1.201879943	0.05235102
O21/ O10	0.72865746	0.050171357
O21/ O11	1.135853999	0.041158001
O21/ O12	0.967606818	0.053555416
O21/ O13	1.116770325	0.181416496
O21/ O14	0.802873342	0.13404053
O21/ O15	1.331110644	0.144996111
O21/ O16	0.840244108	0.113073088
O21/ O17	0.77432568	0.048763178
O21/ O23	2.35188518	0.102447179

Primer Pair	Normalized Interaction Frequencies	Normalized Standard Error of the Mean
sir2-345 HMR Fixed Primer Experiment		
O21/ O1	0.350195017	0.040631129
O21/ O4	0.34841828	0.076433149
O21/ O7	0.658607657	0.143501476
O21/ O8	0.466298561	0.112201503
O21/ O9	0.424374848	0.088471847
O21/ O10	0.280329853	0.023450305
O21/ O11	0.922114093	0.071166059
O21/ O12	0.861292001	0.212553071
O21/ O13	0.792686128	0.130945273
O21/ O14	0.387121836	0.046332101
O21/ O15	0.702583914	0.152852068
O21/ O16	0.587620915	0.086405712
O21/ O17	0.4291798	0.041730262
O21/ O23	2.185230795	0.18254815
